# Synthesis Methods and Therapeutic Journey of Carprofen and Its Derivatives: A Review

**DOI:** 10.1111/cbdd.70122

**Published:** 2025-05-10

**Authors:** Carmen Limban, Diana Camelia Nuță, Miron Teodor Caproiu, Denisa Elena Dumitrescu, Șerban Iancu Papacocea, Alexandra Teodora Bordei, Florea Dumitrașcu

**Affiliations:** ^1^ Department of Pharmaceutical Chemistry, Faculty of Pharmacy “Carol Davila” University of Medicine and Pharmacy Bucharest Romania; ^2^ “C.D. Nenitzescu” Institute of Organic and Supramolecular Chemistry of Romanian Academy Bucharest Romania; ^3^ Department of Organic Chemistry, Faculty of Pharmacy “Ovidius” University of Constanta Constanta Romania; ^4^ Faculty of Medicine, “Carol Davila” University of Medicine and Pharmacy Bucharest Romania; ^5^ Constanta County Ambulance Service Constanta Romania

**Keywords:** antibiofilm, antimicrobial, carbazole, carprofen, drug repurposing, synthesis, veterinary anti‐inflammatory activity

## Abstract

Carprofen, a nonsteroidal anti‐inflammatory drug (NSAID) derived from propanoic acid, is known for its analgesic and antipyretic properties. Although it has long been employed in veterinary medicine as an anti‐inflammatory agent, its use in humans was discontinued shortly after its market launch due to costly raw materials, complex synthesis, and labor‐intensive production processes—factors that made it less competitive compared with other NSAIDs. Despite this, the carprofen molecule remains a subject of significant scientific interest. Recent advancements in its synthesis have introduced simplified and more cost‐effective methods, reigniting its potential for both novel applications and drug repurposing. Exciting new research is exploring carprofen's broader therapeutic possibilities, extending beyond its original anti‐inflammatory role. Studies are investigating its efficacy in antimicrobial therapy—including antibiofilm, anticancer, antiviral, and anti‐Alzheimer's applications—opening doors to a wealth of untapped possibilities. This review delves into these emerging areas, highlighting how carprofen's molecular structure and derivatives can be leveraged to expand its therapeutic reach. The literature review was conducted using four databases: Web of Science, ScienceDirect, Scopus, Embase, and Reaxys. The review focused on English‐language original research and review articles, examining carprofen and its derivatives in terms of their synthesis methods as well as their use as small molecules in various therapeutic applications, both human and veterinary. With ongoing research pushing the boundaries of its potential, carprofen remains a promising candidate for innovation in drug development and treatment strategies.

## Graphical abstract

Although it is currently only used in veterinary therapy, carprofen proves to be a molecule with a versatile therapeutic potential for human pharmacotherapy. Moreover, the molecule is a target in the drug discovery process for the development of new bioactive compounds.

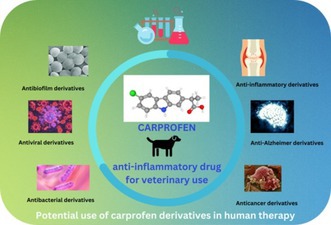

## 1 Introduction

**FIGURE 1:**
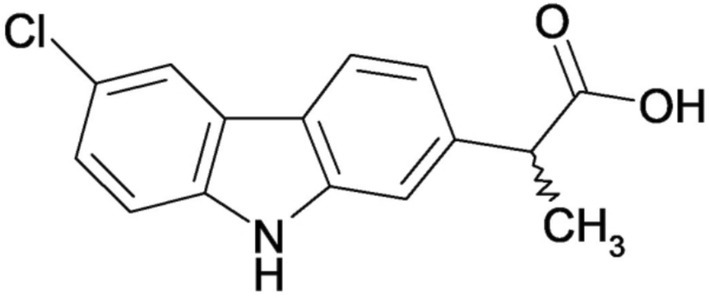
Chemical structure of carprofen.

Carprofen (Figure 1) is a nonsteroidal anti‐inflammatory drug derived from propanoic acid, specifically 2‐(6‐chloro‐9*H*‐carbazol‐2‐yl)propanoic acid according to the IUPAC nomenclature. This compound, which exhibits anti‐inflammatory, analgesic, and antipyretic properties, was initially utilized in human medicine but was subsequently withdrawn from the market due to commercial considerations. Currently, carprofen finds extensive application in veterinary medicine, where it is commonly prescribed as an anti‐inflammatory drug.

The chemical structure of carprofen includes the carbazole nucleus, an important pharmacophore for molecules with anti‐inflammatory, antibacterial (including antituberculosis activity), antifungal, antiviral, antitumor, antiepileptic, antidiabetic, antioxidative, and neuroprotective effects (Kasim et al. 2022; Tiwari et al. 2024). In recent years, for the design of new antimicrobial molecules, the carbazole nucleus has been the subject of the MTDLs (Multi‐Target Direct Ligand) strategy, which is based on the combination of two or more bioactive pharmacophores into a single molecule, resulting in hybrid molecules (Kumar et al. 2023).

Within the molecular structure of carprofen, there exists an asymmetric carbon atom in the side chain, which is a fragment derived from propanoic acid. This structural feature results in two enantiomers, denoted as *S* and *R* forms. The racemic mixture of these enantiomers is employed in therapeutic applications.

It is a white crystalline compound, soluble in ethanol, practically insoluble in water at 25°C.

Long synthesis steps, strict reaction conditions, and expensive raw materials were the reasons for carprofen's withdrawal from human administration.

Carprofen is a nonsteroid anti‐inflammatory drug with selective inhibition of COX‐2 versus COX‐1 (DrugBank 2025). It has also been demonstrated that carprofen is a multitarget‐directed ligand that simultaneously inhibits cyclooxygenases and fatty acid amide hydrolase (FAAH) (Favia et al. 2012).

The carprofen molecule has remained in the attention of researchers, who have conducted drug repurposing or structural modification studies, with the aim of discovering new therapeutic actions for carprofen or obtaining new derivatives with superior pharmacokinetic properties and low toxicity.

The studies conducted on carprofen have highlighted the versatile biological profile of this molecule, including its antibacterial activity against the *Escherichia coli* strain, inhibition of efflux pump activity in *Mycobacterium tuberculosis* , antibiofilm activity on mycobacterial biofilm, inhibition of the main protease of SARS‐CoV‐2, and its antitumor potential through activation of the p38 mitogen‐activated protein kinase.

A recent review emphasized research on the antimicrobial and antibiofilm potential of carprofen as a repurposed drug, noting that NSAIDs have a distinct antimicrobial mechanism of action, allowing them to combat microbial resistance (Kumar and Lavhale 2024).

## 2 Methods

A comprehensive literature review was performed using four prominent scientific databases: Web of Science, ScienceDirect, Scopus, and Reaxys. The search was conducted with a combination of key terms: (“carprofen” OR “carprofen derivative”) AND (“synthesis” OR “pharmacology” OR “biological activity”), covering publications from 1979 to 2024. Only English‐language studies, including both original research and review articles, were considered. The review focused specifically on carprofen and its derivatives, with an emphasis on synthesis methods and their application as small molecules in therapeutic contexts, spanning both human and veterinary medicine.

The selection process involved several systematic steps: initially, duplicates were removed, followed by title and abstract screening to exclude irrelevant studies. Full‐text articles were then assessed for eligibility based on predefined criteria. Articles of the “case study” or “clinical report” type, focused exclusively on clinical data and not addressing chemical aspects, synthesis, or molecular development, were excluded. Articles that did not present concrete experimental data, as well as those that referred to the same topics in a redundant manner without providing additional information compared with those covered in this study, were also excluded.

## 3 Results

### 3.1 Synthesis Methods of Carprofen

**FIGURE 2:**
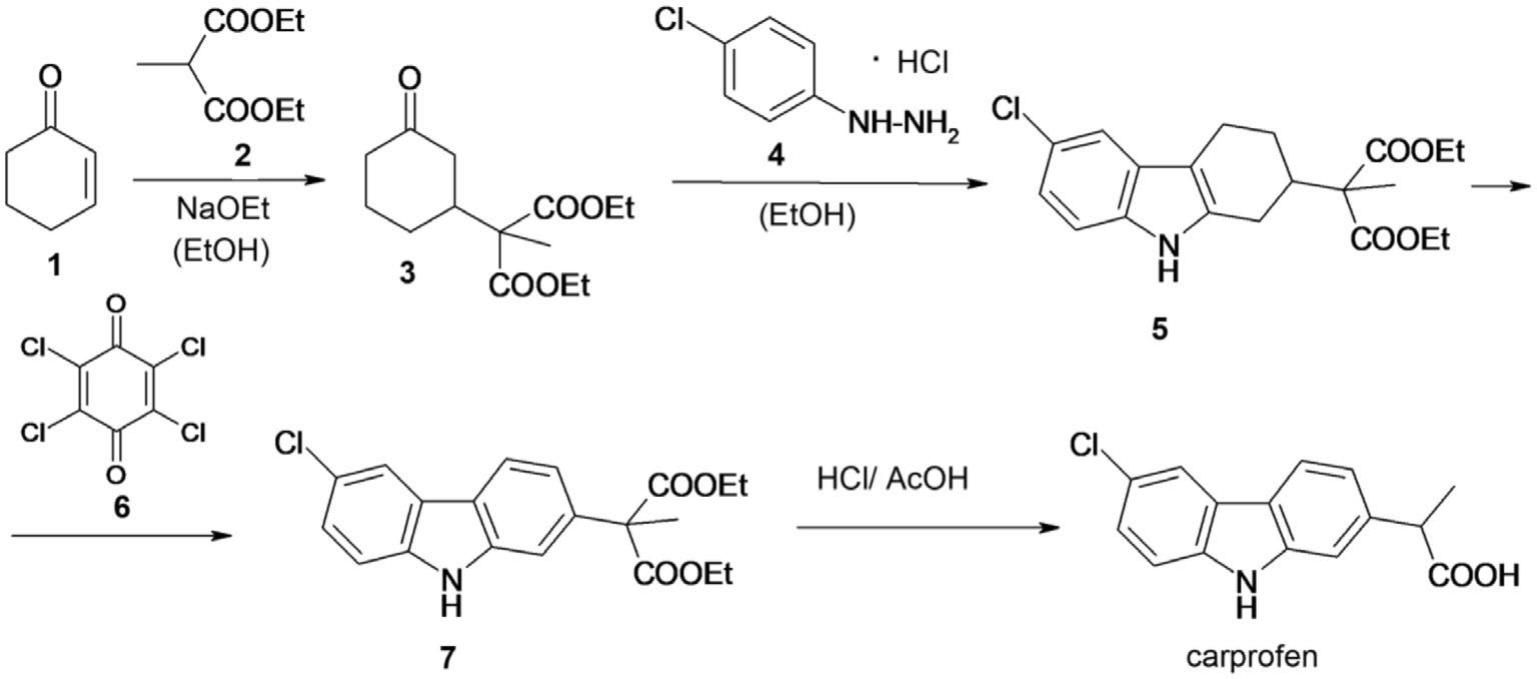
Scheme 1 for the synthesis of carprofen.

Carprofen was first synthesized in 1979 according to Figure 2 (Gurien and Teitel 1979). The process involves the reaction of 2‐cyclohexen‐1‐one (1) with diethyl methyl malonate (2), resulting in the formation of *α*‐methyl‐3‐oxocyclohexane malonic acid diethyl ester (3). Subsequently, compound 3, via the Fischer indolization method, is subjected to treatment with *p*‐chlorophenylhydrazine hydrochloride (4), leading to the synthesis of diethyl‐(6‐chloro‐1,2,3,4‐tetrahydro‐2‐cabazolyl)methyl malonate (5). For aromatization, the next step involves the treatment of compound 5 with chloranil (6), resulting in the production of diethyl‐6‐chloro‐2‐carbazolylmethyl malonate (7). Compound 7 is then subjected to reflux with glacial acetic acid (AcOH) and hydrochloric acid under nitrogen to yield carprofen by a decarboxylation reaction.

However, it is important to note that the raw material, cyclohexenone, utilized in this process is expensive. Consequently, this method of synthesis is deemed unsuitable for the large‐scale industrial production of carprofen.

Other synthesis methods use carbazole as a raw material (Berger et al. 1979) or a derivative thereof (Zwahlen 1981).

**FIGURE 3:**
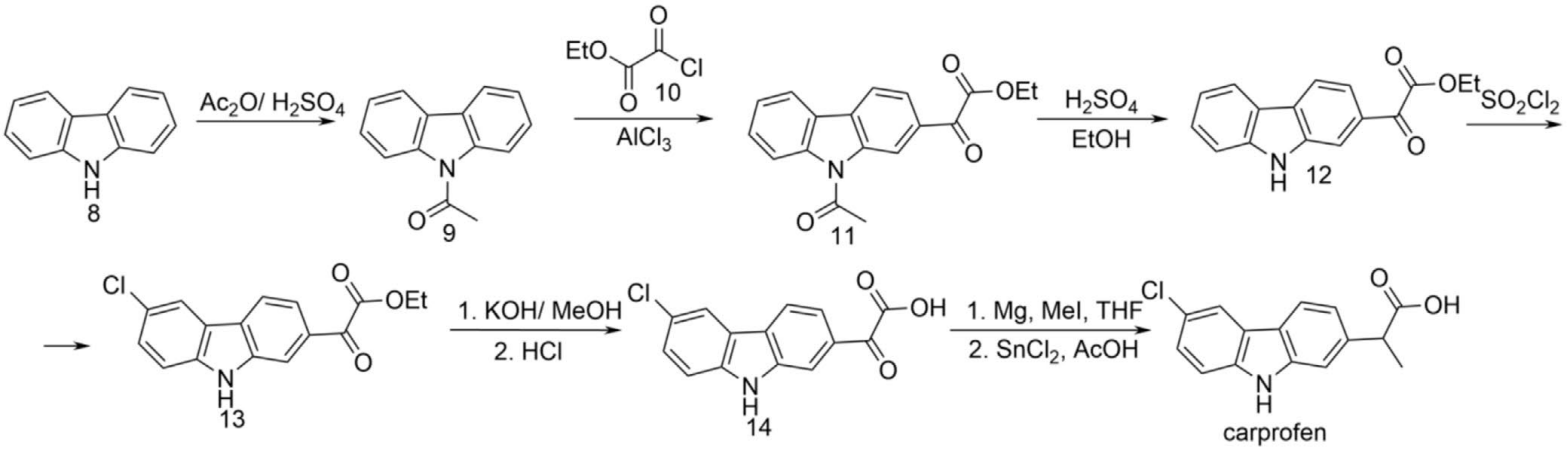
Scheme 2 for the synthesis of carprofen.

Thus, the carbazole (8) is treated with acetic anhydride in sulfuric acid, and the resulting 1‐(9*H*‐carbazol‐9‐yl)ethanone (9) as an intermediate, by treatment with ethyl 2‐chloro‐2‐oxoacetate (ethyl 2‐chloro‐2‐oxoacetate, 10), in the presence of aluminum chloride, forms ethyl 2‐(9‐acetyl‐9*H*‐carbazol‐2‐yl)‐2‐oxoacetate (11) by a Friedel‐Crafts acylation. In the presence of sulfuric acid, compound 11 forms ethyl 2‐(9*H*‐carbazol‐2‐yl)‐2‐oxoacetate (12). This intermediate, reacted with sulfuryl chloride, gives ethyl 2‐(6‐chloro‐9*H*‐carbazol‐2‐yl)‐2‐oxoacetate (13), which is treated with potassium hydroxide in methanol and then the resulting intermediate, with hydrochloric acid. This results in 2‐(6‐chloro‐9*H*‐carbazol‐2‐yl)‐2‐oxoacetic acid (14), which undergoes a Grignard reaction, followed by the reaction of the resulting intermediate with SnCl_2_, finally giving carprofen (Figure 3) (Berger et al. 1985).

This synthesis process is also unsuitable for large‐scale production due to the multistep synthesis and stringent reaction conditions. Carbazole is also an expensive raw material.

**FIGURE 4:**
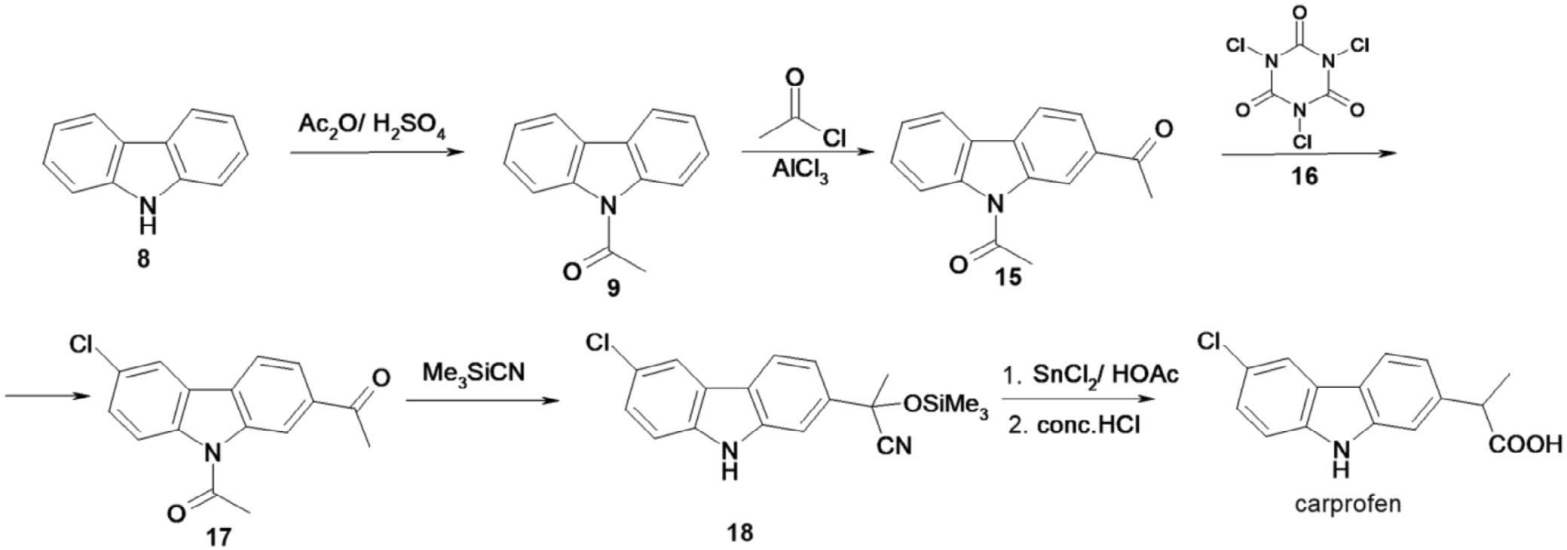
Scheme 3 for the synthesis of carprofen.

Researchers at Hoffmann‐La Roche Inc. presented in 1985 what they considered to be a better process for the preparation of carprofen, also starting from carbazole, as summarized in Figure 4 (Coffen and Mandeville 1987).

In this preparation method, carbazole (8) reacts with acetic anhydride in the presence of sulfuric acid, resulting in 1‐(9*H*‐carbazol‐9‐yl)ethanone (9), which in the presence of acetyl chloride (Friedel‐Crafts acylation) forms 1,1′‐(9*H*‐carbazole‐2,9‐diyl)diethanone (15). This reacts with 1,3,5‐trichloro‐1,3,5‐triazinane‐2,4,6‐trione (trichloroisocyanuric acid, 16) for regioselective chlorination at C‐6, forming 1,1′‐(6‐chloro‐9*H*‐carbazole‐2,9‐diyl)diethanone (17), which by cyanide addition with trimethylsilyl cyanide forms 2‐(6‐chloro‐9*H*‐carbazol‐2‐yl)‐2‐((trimethylsilyl)oxy)propionitrile (18). This intermediate, after treatment with stannous chloride in acetic acid, followed by in situ hydrolysis with concentrated hydrochloric acid, leads to obtaining carprofen.

The method is unsuitable for industrial production due to the necessity of employing a hazardous substance, trimethylsilyl cyanide, and the formation of isomers during both the chlorination step and the subsequent phase.

**FIGURE 5:**
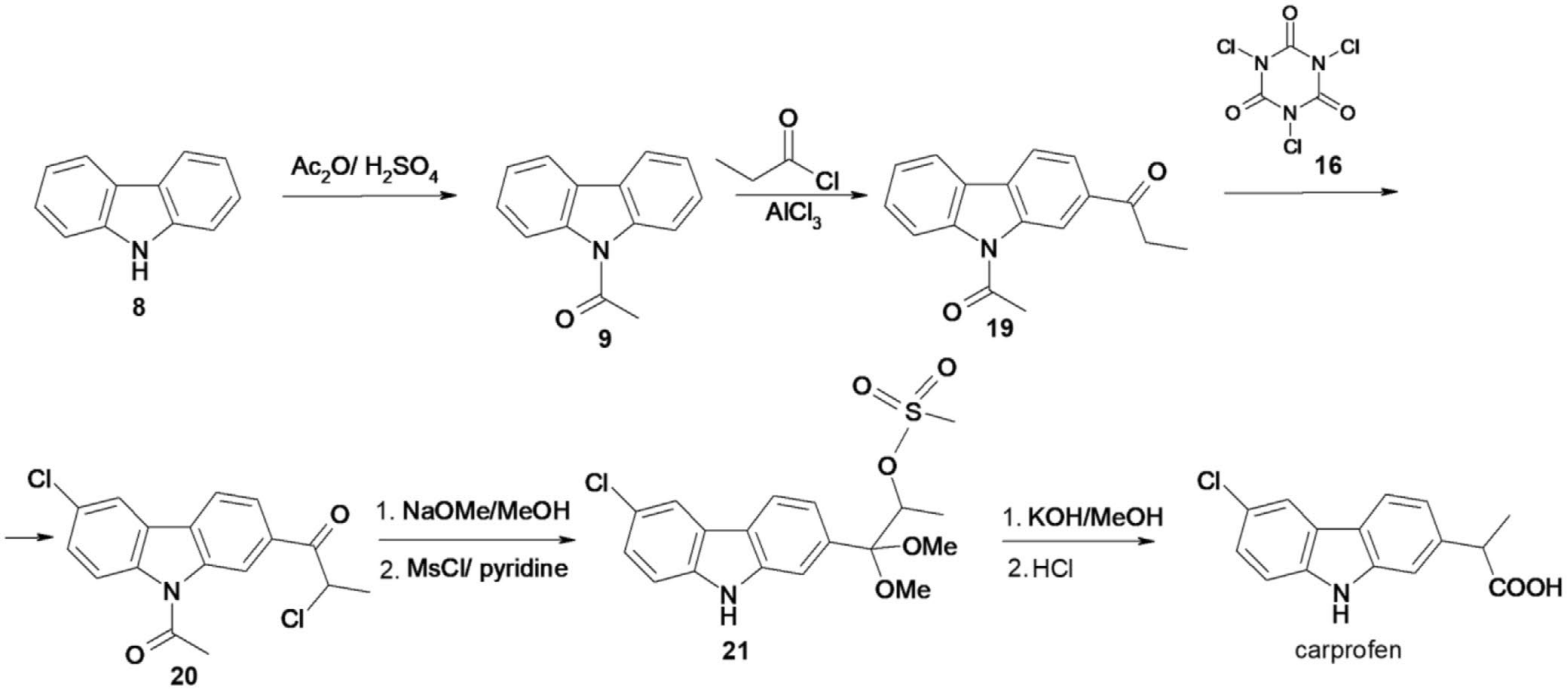
Scheme 4 for the synthesis of carprofen.

Also, Hoffmann‐La Roche researchers reported an improved process for obtaining carprofen and its derivatives, also starting from carbazole, according to the scheme of Figure 5 (Coffen and Mandeville 1987).

After the *N*‐acetylation of carbazole, by a Friedel‐Crafts acylation, the intermediate 1‐(9*H*‐carbazol‐9‐yl)ethanone (9) reacts with propionyl chloride and yields 1‐(9‐acetyl‐9*H*‐carbazol‐2‐yl)propanone (19). The subsequent reaction with trichloroisocyanuric acid (16) produces 1‐(9‐acetyl‐6‐chloro‐9*H*‐carbazol‐2‐yl)‐2‐chloro‐propanone (20). Intermediate 20 undergoes a two‐step reaction, first with sodium methoxide in methanol and then with methanesulfonyl chloride in pyridine, resulting in 1‐(6‐chloro‐9*H*‐carbazol‐2‐yl)‐1,1‐dimethoxypropan‐2‐methanesulfonate (21) in a modified Favorskii rearrangement. To complete the carprofen synthesis, the obtained methanesulfonate is treated with potassium hydroxide in methanol, followed by reaction with hydrochloric acid on the formed intermediate.

This chemical process eliminates the need for toxic trimethylsilyl cyanide. However, the synthesis procedure has its challenges, particularly in the chlorination step, which proves intricate due to the requirement for selective chlorination at specific positions on the molecule, resolved by using trichloroisocyanuric acid (16).

**FIGURE 6:**
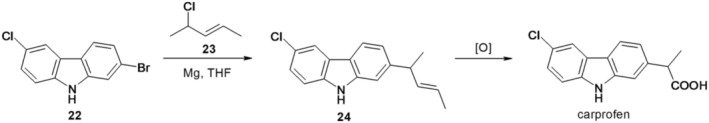
Scheme 5 for the synthesis of carprofen.

Applying the reaction of a Grignard reagent with an allylic halide in a dipolar aprotic solvent (THF), 2‐bromo‐6‐chloro‐9*H*‐carbazole (22) undergoes a coupling reaction with 4‐chloro‐2‐pentene (23), giving 6‐chloro‐2‐(pent‐3‐en‐2‐yl)‐9*H*‐carbazole (24). This intermediate was oxidized (see Figure 6) and the carprofen was obtained (Yi et al. 2016; Yi et al. 2017).

However, this procedure is deemed impractical for the industrial production of carprofen due to the involved raw material, 7‐chloro‐3‐bromocarbazole, which necessitates several expensive synthesis steps.

**FIGURE 7:**
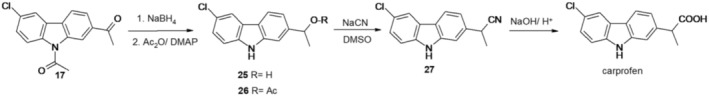
Scheme 6 for the synthesis of carprofen.

In an alternative method, 1′‐(6‐chloro‐9H‐carbazole‐2,9‐diyl)diethanone (17) underwent a series of transformations. Initially, it was subjected to sequential reduction with sodium borohydride, followed by acetylation of the resulting alcohol derivative (25) using acetic anhydride, leading to the formation of the acetate (26). The ester is cleaved using sodium cyanide in an aprotic solvent (DMSO) at 130°C–135°C, to yield the corresponding nitrile analogue (27). LiCN in DMF at 50°C can also be used. The final step involved the hydrolysis of compound 27 using NaOH in ethylene glycol at 180°C–185°C for 2.5 h or in refluxing methanol for 24 h, ultimately yielding carprofen (Figure 7) (Manchand et al. 1994).

Lin Yi et al. have developed new procedures for the preparation of carprofen and its derivatives (Yi et al. 2016, 2017).

**FIGURE 8:**
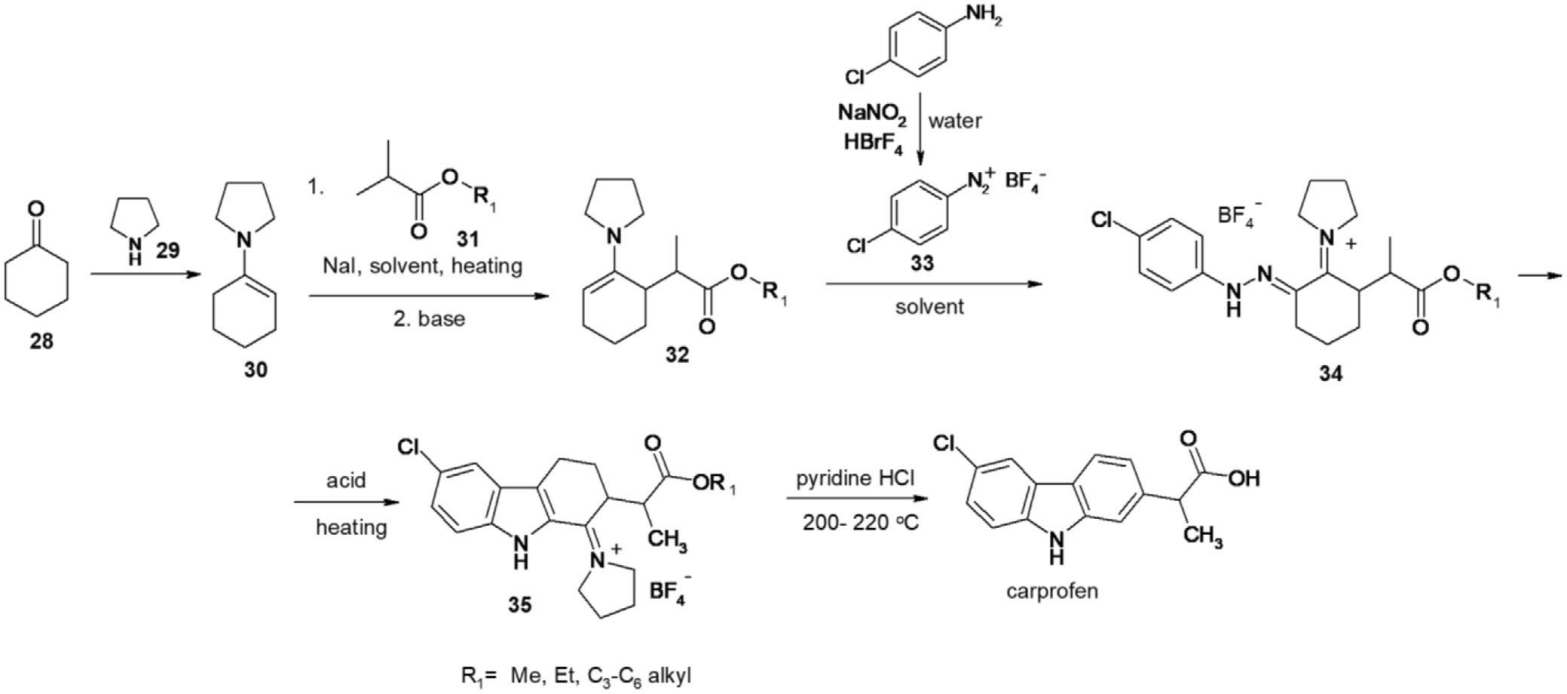
Scheme 7 for the synthesis of carprofen.

In this context, the process involved heating cyclohexanone (28) and pyrrolidine (29) with toluene sulfonic acid as a catalyst, leading to the formation of 1‐(cyclohex‐1‐en‐1‐yl)pyrrolidine (30). The enamine (30) subsequently reacts with the ester of an *α*‐substituted carboxylic acid (31) upon heating in an organic solvent, such as acetonitrile, utilizing sodium iodide as a catalyst. After treatment with a base, such as sodium methoxide, alkyl 2 (2‐pyrrolidin‐1yl)cyclohex‐2‐en‐1‐yl‐propanoate (32) is produced. The 2‐substituted cyclohexanone enamine 32 is then subjected to treatment with 4‐chlorophenyldiazonium tetrafluoroborate (33) in tetrahydrofuran, resulting in the formation of 1‐(2‐(2‐(4‐chlorophenyl)hydrazono)‐6‐(1‐methoxy‐1‐oxopropan‐2‐yl)cyclohexzlidene)pyrrolidin‐1‐ium tetrafluoroborate (34). Compound 33 is obtained by treating 4‐chloroaniline with sodium nitrite and tetrafluoroboric acid in the presence of an acid solution at room temperature. The derivative 34 is converted to 1‐(6‐chloro‐2‐(1‐alkoxy‐1‐oxopropan‐2‐yl)‐2,3,4,9‐tetrahydro‐1*H*‐carbazol‐1‐ylidene)pyrrolidin‐1‐ium tetrafluoroborate (35) under Fisher indole conditions by refluxing in methanol and in the presence of polyphosphoric acid. Carprofen is obtained by heating compound 35 with pyridinium hydrochloride (Figure 8).

**FIGURE 9:**
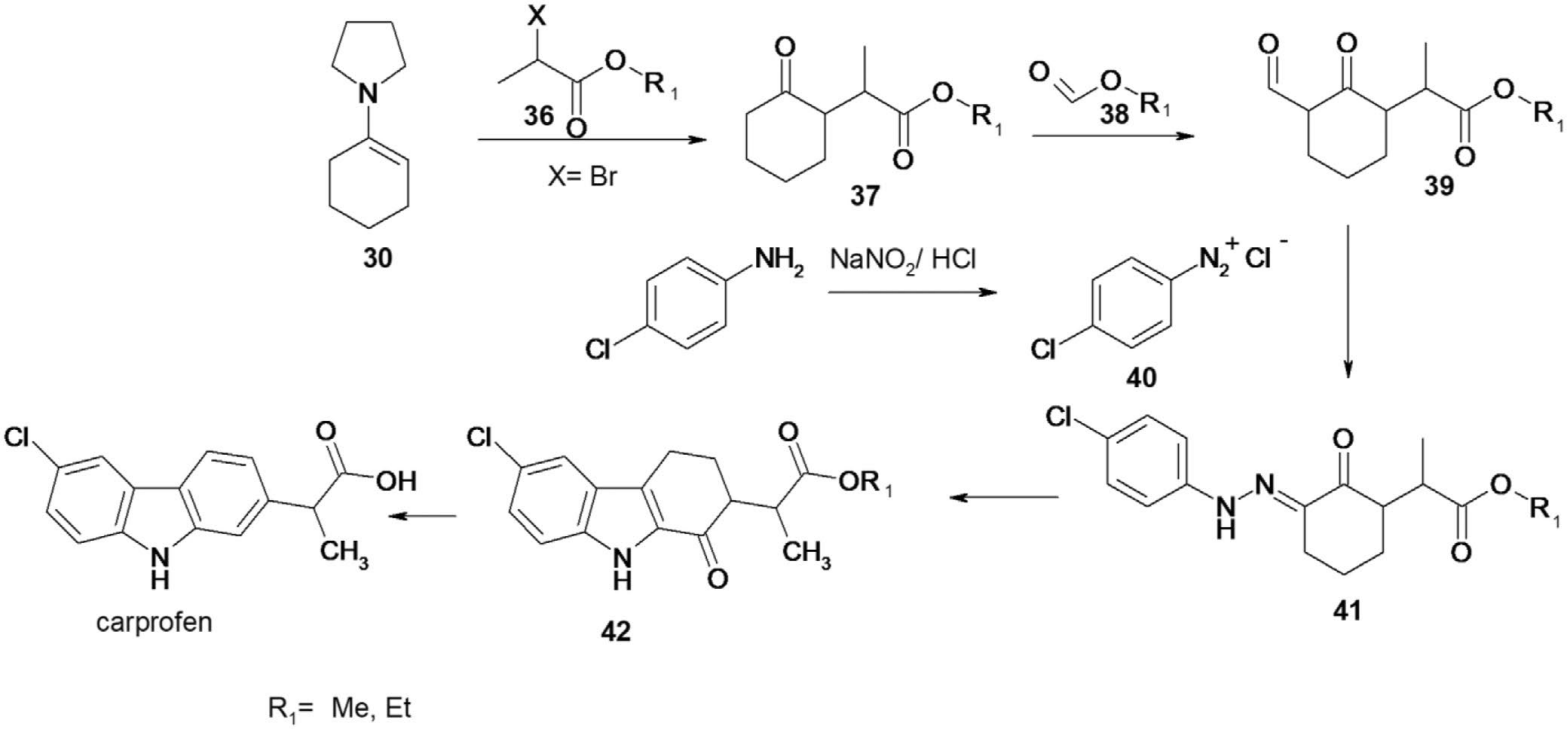
Scheme 8 for the synthesis of carprofen.

The steps of an alternative carprofen synthesis variant, offering a simplified, cost‐effective, efficient, and viable process suitable for industrial‐scale manufacturing, are illustrated in Figure 9 and comprise the following stages:

- The reaction of enamine 30 with an ester of an α‐substituted carboxylic acid (ethyl 2‐bromopropanoate, 36), results in a substituted 2‐cyclohexanone, alkyl 2‐(2‐oxocyclohexyl)propanoate (37).
- The reaction of 2‐substituted cyclohexanone with a formic acid ester (38), in the presence of sodium methoxide, leads to the formation of alkyl 2‐(3‐formyl‐2‐oxocyclohexyl)propanoate (39).
- The reaction of compound 39 with an aqueous solution of a substituted phenyldiazonium salt (40) to produce alkyl 2‐(3‐(2‐(4‐chlorophenyl)hydrazono)‐2‐oxocyclohexyl)propanoate (41). The substituted phenyldiazonium salt is prepared from 4‐chloroaniline and sodium nitrite in hydrochloric acid.
- The heating of compound 41 in the presence of hydrochloric acid results in the preparation of the compound alkyl 2‐(6‐chloro‐1‐oxo‐2,3,4,9‐tetrahydro‐1*H*‐carbazol‐2‐yl)propanoate (42).
- The heating of compound 42 in the presence of pyridine hydrochloride yields carprofen.

**FIGURE 10:**
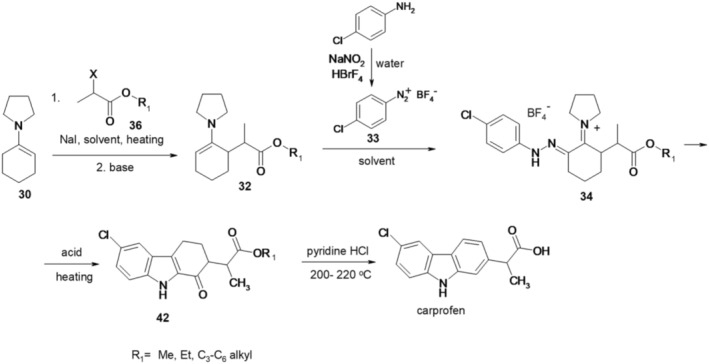
Scheme 9 for the synthesis of carprofen.

The production of carprofen is replicated in another synthesis variant, akin to the two previously outlined methods, with its respective steps depicted in Figure 10.

Grob fragmentation is an excellent method for the formation of medium or large rings from bicyclic systems. This can also be applied to the synthesis of carprofen (Shi et al. 2017).

**FIGURE 11:**
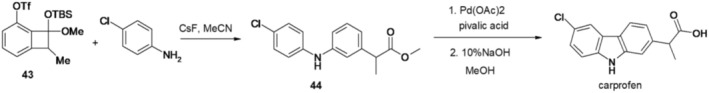
Scheme 10 for the synthesis of carprofen.

The electron‐withdrawing nature of the trifluoromethanesulphonate (OTf) group within the structure of compound 43 facilitates the initiation of the tetraatomic cycle's opening, resulting in the creation of an intermediate. In the presence of 4‐chloroaniline, this intermediate transforms into compound 44, subsequently progressing to the methyl ester of carprofen. Ultimately, through hydrolysis, carprofen is formed (Figure 11).

**FIGURE 12:**
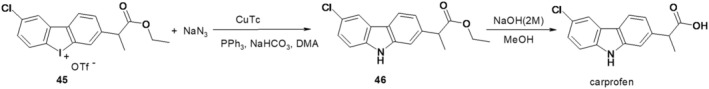
Scheme 11 for synthesis of carprofen.

Also, the carbazole nucleus was obtained by nitrogen–iodine exchange between a diaryliodonium salt and sodium azide as a nitrogen source. Thus, carprofen was efficiently synthesized from a diaryliodonium salt 45, using copper(I) thiophene‐2‐carboxylate (CuTc) as a catalyst, in the presence of triphenylphosphine (PPh_3_) and in a basic environment, followed by a hydrolysis step of the intermediate 46 result (Figure 12) (Wang et al. 2018).

An alternative approach to obtaining carprofen starts with 4‐bromophenyl acetic acid (47), which undergoes esterification with methanol and a catalytic amount of thionyl chloride, yielding its methyl ester 48. Treatment of compound 48 with lithium diisopropylamide (LDA) and methyl iodide results in the formation of methyl 2‐(4‐bromophenyl)propionate (49) (Kumar Goud Bhatthula et al. 2019).

Compound 49 undergoes a reaction with bis(pinacolato)diboron and [1,1′‐bis(diphenylphosphino)ferrocene]‐dichloropalladium (Pd(dppf)Cl2), potassium acetate in 1,4‐dioxane, producing methyl 2‐[4‐(4,4,5,5‐tetramethyl‐[1,3,2]dioxaborolan‐2‐yl)phenyl]propionate (50). The Suzuki‐Miyaura cross‐coupling technique, a palladium‐catalyzed reaction between organic boron compounds and organic halides, was employed to synthesize methyl 2‐{5′‐chloro‐2′‐nitro‐[1,1′‐biphenyl]‐4‐yl}propionate (51) by coupling phenyl dioxaborolane ester 50 with 2,4‐dichloronitrobenzene.

**FIGURE 13:**
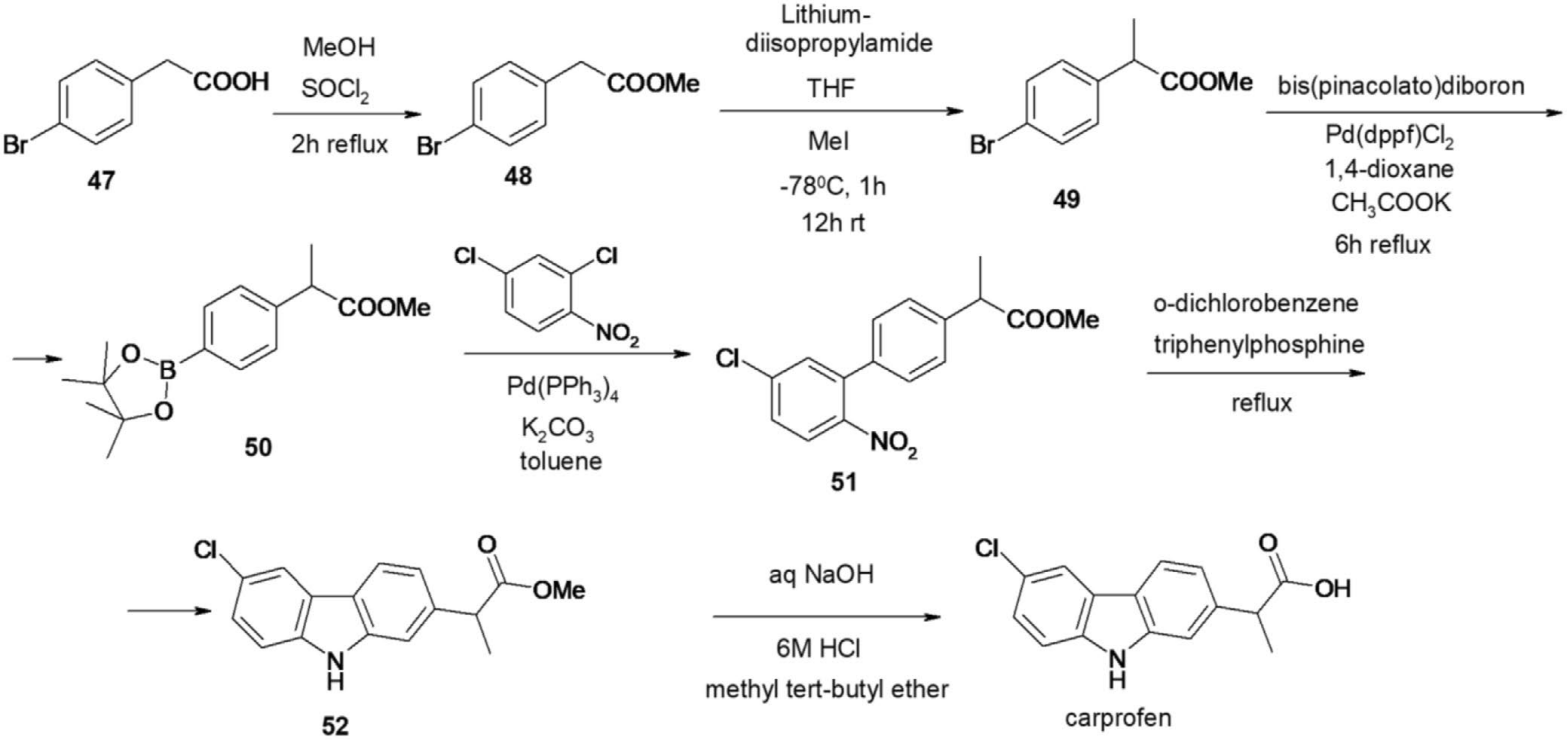
Scheme 12 for the synthesis of carprofen.

The subsequent step involves Cadogan reductive cyclization of 2‐nitrobiphenyl 51, mediated by triphenylphosphine in *o*‐dichlorobenzene, resulting in methyl 2‐(6‐chloro‐9*H*‐carbazol‐2‐yl)propionate (52). Hydrolysis of compound 52 leads to the ultimate formation of carprofen (Figure 13).

Efforts to achieve high yields in carprofen synthesis have also explored the electrochemical method. This alternative approach involved electrochemical hydrogenation, eliminating the need for an external supply of hydrogen gas. Using an undivided cell with a nickel cathode and a lead anode in the presence of sulfuric acid, carprofen was obtained with an advanced level of purity. The compound was easily isolated, and the yields were significantly influenced by substrate concentration, temperature, and current density. The electrochemical reaction employed 6‐chloro‐α‐methyl‐9*H*‐carbazole‐2‐ethene‐1‐oic acid dissolved in acidified ethanol, with a constant current density of 100A/m^2^ (Raju et al. 2003).

**FIGURE 14:**
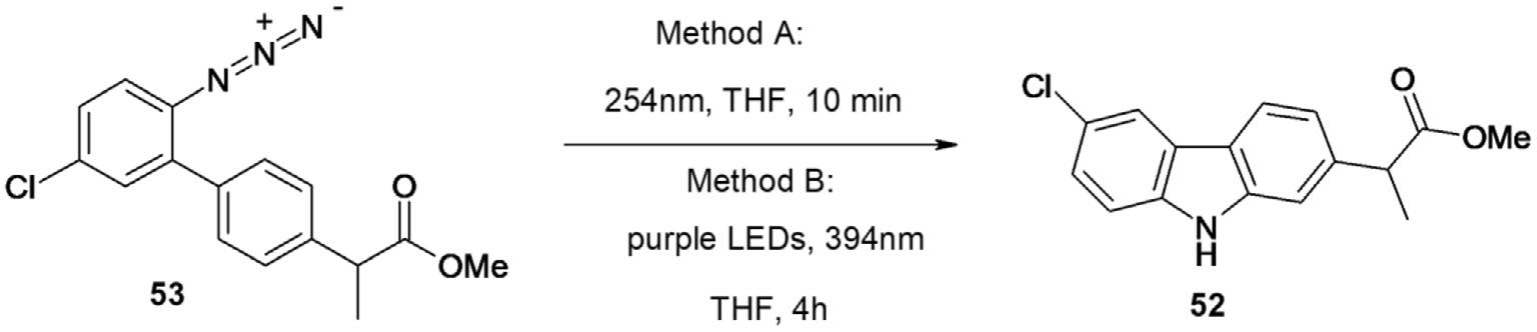
Photochemical routes for synthesis of methyl ester of carprofen.

Polycyclic heterocycles such as carbazole can be obtained by photochemical conversion of appropriately substituted aryl azides in a short reaction time under low energy (394 nm) or violet LED irradiation in a reactor with continuous flow (Parisien‐Collette et al. 2017). The photochemical method is believed to operate through a distinct mechanism compared to other azide activation methods that necessitate transition metal catalysis. Employing azidobiphenyl 53 in the intramolecular photochemical amination process yielded carprofen methyl ester (52). The compound was isolated as the predominant product with a 50% yield at 254 nm and a 64% yield at 394 nm. It was obtained as a single regioisomer, and no dechlorination was observed (Figure 14).

The problems related to the complexity of the chemical synthesis of carprofen can be mitigated by identifying carprofen analogues with a similar potency.

### 3.2 The Pharmacological Action of Carprofen in Humans

Carprofen follows the conventional mechanism of nonsteroidal anti‐inflammatory drugs (NSAIDs), specifically by hindering the synthesis of prostaglandins (PG) through the inhibition of the enzyme cyclooxygenase (COX).

In human usage, it was recommended for approximately a decade, commencing in 1985, to alleviate pain stemming from severe trauma, such as postoperative inflammation, and to manage pain associated with arthritis or inflammation. Following oral administration and metabolism, carprofen is excreted in the urine as the glucuroconjugate derivative, constituting approximately 65%–70% of the elimination. The remaining portion of the compound is eliminated through the bile and enters the enterohepatic circuit. Less than 5% of the administered dose is excreted in the urine as free carprofen. Additionally, there is no preference for any enantiomer during glucuroconjugation (Rubio et al. 1980).

An article dating back to 1989 (Lindenmuth et al. 1989) provides an assessment of the carprofen molecule, which was a novel substance at that time. Described as a nonsteroidal anti‐inflammatory drug (NSAID) with documented analgesic and antipyretic effects, carprofen stood out for its noteworthy half‐life of 10–12 h and the absence of gastrointestinal adverse effects—a rarity during that period. The study aimed to compare carprofen with other anti‐inflammatories, establishing its safety and efficacy. Conducted as a double‐blind, randomized trial involving 248 patients aged 15–63 years, the participants were divided into five groups. They received carprofen in three concentrations, along with diflunisal and aspirin, with an additional group receiving a placebo. The response to a single dose of each drug was assessed based on self‐reported pain intensity and improvement, while adverse reactions were monitored and evaluated for each case. Notably, side effects, particularly those affecting the nervous system (primarily drowsiness) and the digestive system (such as diarrhea), were observed. Diflunisal exhibited the highest rate of adverse reactions compared to aspirin and carprofen, with carprofen having the lowest percentage of adverse reactions (22.6%). This clinical study portrayed carprofen as an effective substance, appreciated for its lack of adverse gastrointestinal effects.

The ongoing evaluation of this molecule has extended to the exploration of potential side effects, including photoallergic contact dermatitis, which has been reported in individuals exposed to carprofen. Due to its potency as a photoallergen, special care is warranted in handling carprofen (Kerr et al. 2008).

### 3.3 The Pharmacological Action of Carprofen in Veterinary

While NSAIDs have consistently drawn heightened scrutiny for their adverse reactions in human therapy, their utilization in veterinary medicine has posed fewer challenges. Adverse effects in animals have frequently been milder or even nonexistent, owing to variations in metabolism and the mechanism of action of these anti‐inflammatory agents across different species (Fox and Johnston 1997).

Carprofen is currently approved for veterinary use in muscle pain, surgical and posttraumatic pain, mastitis, osteoarthritis, and various respiratory diseases in dogs and cats, but can also be administered to horses, cattle, sheep, rabbits, rodents, and other mammals, small birds, and reptiles (Plumb 2018).

Carprofen has obtained approval from the Food and Drug Administration for the treatment of pain and inflammation linked to osteoarthritis, as well as acute pain following orthopedic and soft tissue surgeries in dogs. Additionally, in the EU and various other countries, carprofen has been sanctioned for application in other animal species (MSD Veterinary Manual 2024).

Carprofen has an anti‐inflammatory effect similar to indomethacin, diclofenac, and piroxicam, having a stronger anti‐inflammatory effect than aspirin, ibuprofen, and phenylbutazone, while being safer than many other NSAIDs. Analgesic and antipyretic actions have been shown to be similar to indomethacin and stronger than phenylbutazone or aspirin (Fox and Johnston 1997).

Studies conducted in dogs indicate that this medication selectively blocks COX‐2, with minimal impact on COX‐1 (Plumb 2018). The selective character has the advantage of reducing the risk of gastrointestinal and renal adverse reactions, while maintaining its effectiveness in reducing inflammation.

In dogs, the oral bioavailability of carprofen is high (90%), and plasma concentrations are reached approximately 2–3 h after administration (MSD Veterinary Manual 2024). When given subcutaneously at a dosage of around 2 mg/kg body weight, carprofen exhibits slower absorption compared to oral administration. Nevertheless, the overall absorption of the drug within the initial 12 h after a single dose, or during steady‐state conditions for repeated doses, appears to be comparable between the two routes of administration (Zoetisu 2019).

The volume of distribution is limited, thought to be constrained by high plasma protein binding. The volume of distribution of carprofen in different animal species is shown in Table 1 (Zoetisus 2019; Riviere et al. 2009).

**TABLE 1:** Volume of distribution of carprofen (racemic and enantiomers) in different animal species.

Species	Volume of distribution		
	Racemic mixture	R(−)	S(+)
Dog	0.14 ± 0.02 L/kg	0.12 ± 0.03 L/kg	0.19 ± 0.05 L/kg
Cat	0.15 ± 0.04 L/kg	0.24 ± 0.05 L/kg	0.35 ± 0.10 L/kg
Horse	0.22 ± 0.01 L/kg	0.10 ± 0.01 L/kg	0.29 ± 0.04 L/kg
Sheep	0.093 ± 0.006 L/kg	—	—
Rat	—	0.228 ± 0.043 L/kg	0.242 ± 0.030 L/kg

The half‐life differs from species to species and is listed in Table 2 (Zoetisu 2019; Riviere et al. 2009).

**TABLE 2:** Half‐life of caprofen (racemic and enantiomers) in different species.

Species	*T*½ (hours)		
	Racemic mixture	R(−)	S(+)
Dog	8.00 ± 1.18 h or 11.7 ± 3.05 h	—	—
Cat	19.4 ± 7.25 h or 20.0 ± 16.6 h	21.3 ± 9.09 h	14.6 ± 5.78 h
Horse	21.9 ± 2.3 h or 18.1 ± 1.3 h	18.36 ± 1.02 h or 20.6 ± 2.55 h	9.86 ± 1.29 h or 16.8 ± 1.77 h
Sheep	26.1 ± 1.14 h	—	—

Similar to other NSAIDs, carprofen exhibits high protein binding, estimated at around 99%. Its duration of action was evaluated in cats using postoperative analgesia, and it ranged between 18 and 24 h (measured by visual analogue scale) after a subcutaneous dose of 4 mg/kg body weight, given either before surgery or at extubation (Zoetisu 2019; Riviere et al. 2009).

Studies conducted in equine, canine, sheep, and guinea pig species demonstrate that carprofen undergoes hepatic biotransformation. In these species, both R(−) and S(+) enantiomers are metabolized via glucuronidation. However, the rate of metabolism of these enantiomers varies across species. In vitro experimentation reveals that the R‐(−)‐glucuronide‐conjugated enantiomer is present in higher concentrations than the S‐(+)‐enantiomer in liver microsomes. The differing rates of glucuronide conjugation may be attributed to pharmacokinetic variations between the enantiomers.

In horses, the predominant glucuronidation of the *S‐*(+)‐enantiomer may elucidate the higher concentrations of the *R‐*(−)‐enantiomer found in plasma samples. Elimination primarily occurs through hepatic biotransformation, with resulting metabolites excreted in feces and urine.

Delatour et al. found that carprofen has enantioselective pharmacokinetics in calves, the R(−) enantiomer being the predominant form in plasma samples (Delatour et al. 1996).

Research in dogs indicates that 70%–80% of carprofen metabolites are excreted in feces, while only 10%–20% are excreted in urine. These metabolites include a glucuronide‐conjugate ester, as well as two phenolic metabolites: 7‐hydroxycarprofen and 8‐hydroxycarprofen.

In guinea pigs, approximately 60%–75% of an intravenously administered dose is eliminated through biliary secretion and gastrointestinal elimination, with around 20%–30% excreted in urine. Plasma primarily contains the *S*(+) enantiomer, whereas the *R*(−) enantiomer undergoes greater glucuronidation in the liver. Less than 5% of the administered drug dose is eliminated in its free, unmetabolized form (Zoetisu 2019; Riviere et al. 2009; Rubio et al. 1980).

There are oral or injectable forms, administered i.v., i.m., s.c. (Uney et al. 2021).

The recommended dose in dogs is 4.4 mg/kg per day or divided every 12 h, p.o. In dogs with osteoarthritis, carprofen can be used for more than 28 days without serious adverse effects. A single dose is preferred in cats and cattle due to the risk of gastrointestinal hemorrhage (Plumb 2018).

Reported adverse reactions primarily included gastrointestinal symptoms such as vomiting, diarrhea, and gastrointestinal ulceration, with renal and hepatic reactions occurring infrequently. In dogs, rare instances of potentially severe idiosyncratic hepatopathies, marked by acute liver necrosis, have been documented, without any confirmed breed predisposition. It is advisable to conduct clinical laboratory monitoring for signs of liver damage, particularly in geriatric animals who might be more susceptible to severe complications (MSD Veterinary Manual 2024).

A study from 2013 (Monteiro‐Steagall et al. 2013) set out to centralize and re‐evaluate the data available up to that point on the known adverse effects of NSAIDs in dogs, carprofen being one of the most commonly used substances in the canine species. A few side effects reported following carprofen‐based treatment in dogs can be mentioned: liver damage, decreased platelet aggregation rate, hypocoagulability. Their incidence rates were low. At the same time, there are studies in which such reactions were not identified at all. So, the general conclusion of this study supports the hypothesis that the adverse reactions occurring when carprofen is administered as an anti‐inflammatory in animals, and especially in dogs, are small compared with the beneficial effect of the medication.

The use of analgesic medication in fish has been quite rare, due to the belief that they do not have the necessary receptors to perceive pain. After nociceptors were identified in fish, similar to those of mammals, attention increased on the methods of analgesia used in their treatment. Both opioids and nonsteroidal anti‐inflammatory drugs have been used. Opioid medication caused severe side effects in the respiratory and cardiovascular systems, while NSAIDs were free of such effects. In a recent study on juvenile rainbow trout ( *Oncorhynchus mykiss* ), the bioavailability and overall pharmacokinetics of the carprofen molecule were analyzed (Uney et al. 2021).

The assessment of pharmacokinetics holds significant importance as it provides a valuable quantitative foundation for further understanding the behavior of an active substance within the body. Prior to this study, no research had been conducted on the pharmacokinetics of carprofen in fish. The decision to conduct this evaluation stemmed from existing evidence indicating the generally superior pharmacological characteristics of carprofen, such as high bioavailability, extended half‐life, and minimal risk of gastrointestinal side effects.

Following this investigation, specific processes related to absorption, distribution, metabolism, and elimination (ADME) could be quantified, thereby aiding in the determination of safe and efficacious dosage regimens and optimal routes of administration. Intravenous, intramuscular, and oral routes were employed in this study, with carprofen plasma concentrations measured using high‐performance liquid chromatography (HPLC) and pharmacokinetic parameters derived through noncompartmental analysis.

The findings underscored favorable bioavailability and prolonged half‐life when carprofen was administered intramuscularly and orally, suggesting potential effectiveness for these applications in fish. No adverse reactions were observed at a dosage of 2.5 mg/kg via any of the three selected routes. However, the half‐life was found to vary, influenced by ambient temperature, which can impact fish physiology and metabolism.

Regarding the route of administration, intramuscular injection was deemed more suitable for ornamental fish species and fry, while alternative administration methods were recommended for adult individuals. The option of oral administration, particularly when administered with food, was highlighted due to promising pharmacokinetic data associated with this approach.

The initial hypothesis regarding favorable pharmacokinetic properties, characterized by an extended half‐life and high bioavailability, was substantiated. Nonetheless, further studies on the clinical efficacy and safety of carprofen are necessary before considering its use in treating various conditions in juvenile rainbow trout.

In recent years, the study of carprofen has gone in other directions, starting with the idea of discovering new uses or reintroducing it as a modified molecule for human use. In this sense, other potential biological targets of carprofen and its derivatives were searched, using the propanoic acid moiety and the carbazole nucleus as pharmacophores.

### 3.4 Biological Actions of Carprofen Derivatives

The carprofen molecule continues to attract researchers' attention, despite being in existence for several decades. Thus, through molecular modeling studies, compounds with antimicrobial potential, including antibiofilm, anticancer, antiviral, or anti‐Alzheimer's, were obtained.

#### 3.4.1 Carprofen Derivatives With Anti‐Inflammatory Effect

The development of new selective COX‐1 and COX‐2 inhibitors using virtual screening and docking study has been successfully used to design new candidates as more active and safer drugs to overcome the side effects at the gastrointestinal tract of classical NSAIDs. Thus, in a study from 2019 (Gouda and Almalk 2019), four series of carprofen derivatives were designed by isosteric replacement of –NH– in the carbazole nucleus, with –O–, –S–, and –CH2–. Thus, to study the affinity, binding mode, and selectivity to COX1/2, the following were designed: (*S*)‐2‐(9H‐carbazol‐1/2/3/4‐yl)propanoic acid derivatives (26 derivatives), (S)‐2‐(9H‐fuoren‐3‐yl)propanoic acid derivatives (16 derivatives), (*S*)‐2‐(dibenzo[b,d]furan‐3‐yl)propanoic acid derivatives (16 derivatives), (*S*)‐2‐(dibenzo[b,d]thiophen‐3‐yl)propanoic acid derivatives (20 derivatives) and a second series of 22 derivatives, analogs of thiophene having alkyl groups at C_3_, C_4_, and C_8_.

Substitution of chlorine in the 6‐position of carprofen with fluorine, bromine, or iodine resulted in a marked decrease in selectivity towards COX‐1. Among these, the 6‐iodo derivative (54) displayed the highest binding affinity for COX‐2.

**FIGURE 15:**
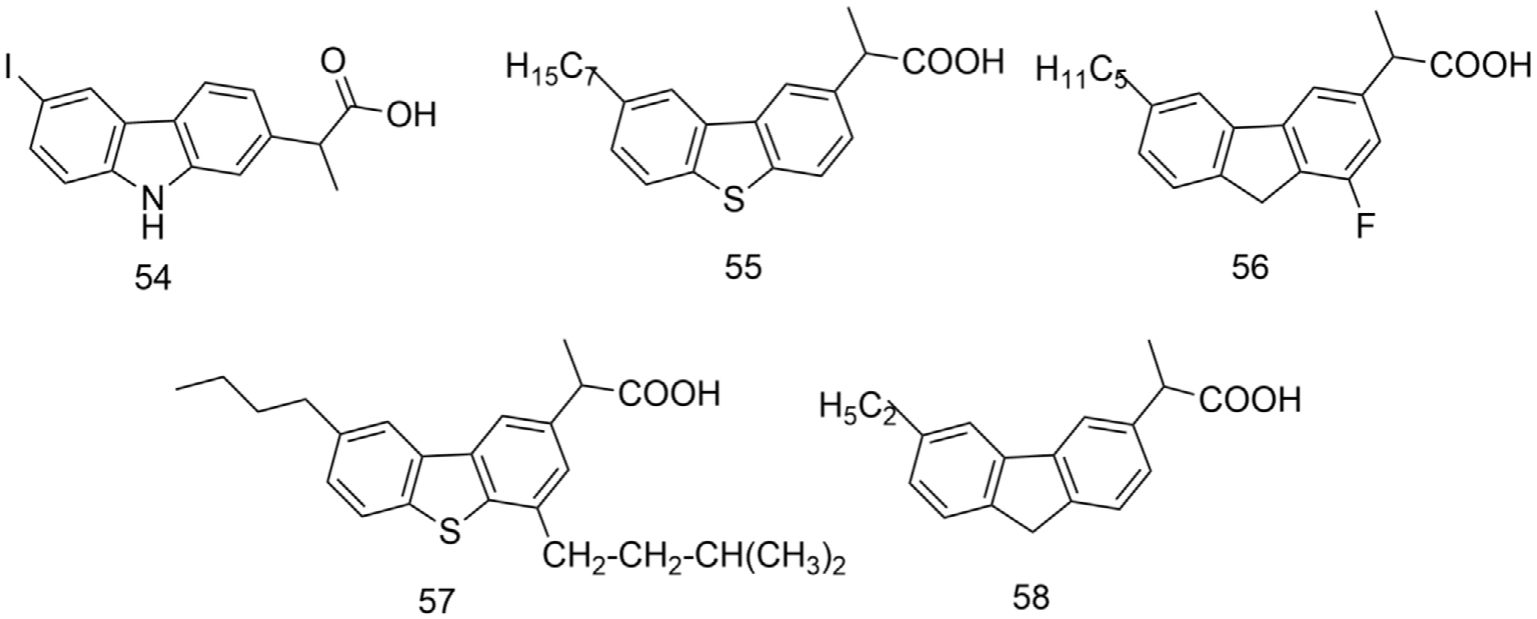
Structural formulas of compounds 54, 55, 56, 57, 58.

The impact of the alkyl groups of the designed analogs on the binding affinity and inhibition constants was evaluated using molecular docking study. The results showed that the position and length of the alkyl substituents greatly influence the binding mode. The introduction of alkyl groups at C1 and C6 of the four series improved the binding to COX‐1, while the presence of alkyl groups at C4 increased the binding affinity of COX‐2. Compound 55 showed the highest binding affinity for COX‐1 and COX‐2, and compounds 56 and 57 showed the highest selectivity potential for COX‐1 and COX‐2, respectively. Drug‐likeness study was performed for the most promising compounds. Thus, derivative 58 showed a drug‐likeness score of 0.96, as compared to 0.30 for *S*‐carprofen (Figure 15). The obtained results highlighted the importance of hydrophobic interactions in modulating the selectivity and binding affinity to COX‐1/2 of the new potential NSAIDs.

Carprofen demonstrated potent inhibition of fatty acid amide hydrolase (FAAH), exhibiting minimal gastrointestinal side effects. These results prompted the synthesis of novel carprofen derivatives aimed at harnessing its anti‐inflammatory properties while mitigating adverse effects (Favia et al. 2012).

It is worth mentioning the context of the discovery of the effect of carprofen as an FAAH inhibitor and the relevance of this discovery. The mechanisms of action of NSAIDs are well known: the inhibition of COX‐1 and COX‐2. Blocking COX‐1 brings with it the anti‐inflammatory effect and a series of adverse reactions at the gastrointestinal level, due to the simultaneous interruption of the synthesis of prostaglandins with the role of gastric protection (PGE2). This is true in the case of nonselective NSAIDs; the selective ones, which act on COX‐2, offer greater safety in terms of gastrointestinal reactions. However, even in the case of COX‐2 inhibitors, more and more evidence is emerging regarding possible adverse cardiovascular effects.

Equally, in recent decades a relatively new category of substances that have an anti‐inflammatory effect—FAAH inhibitors—has appeared. Studies have been carried out that put in a favorable light the combination of the two types of anti‐inflammatory action mechanisms, in a synergism of potentiating the desired therapeutic action, simultaneously with a significant decrease in adverse reactions (Naidu et al. 2009; Sasso et al. 2012). The findings from these investigations have spurred researchers to pursue a new objective: identifying molecules capable of targeting multiple receptors, specifically to inhibit both COX and FAAH. Simultaneous inhibition of these enzymes yields an amplified analgesic response and mitigated side effects in vivo.

In this regard, a study conducted in 2012 by Favia et al. explored structural similarities between enzymes and compounds, as well as topological distances, pinpointing the carprofen molecule as one of the most potent COX/FAAH co‐inhibitors among a pool of 382 evaluated substances. This pool included compounds like indomethacin, flurbiprofen, celecoxib, among others. The mean effective concentrations were recorded as IC50 = 79 μM (FAAH), IC50 = 22 μM (COX‐1), and IC50 = 4 μM (COX‐2).

To delve deeper into this inquiry, compounds derived from structural modeling of the carprofen molecule were synthesized and studied. Modifications involved the removal of the chlorine atom, functionalization of the carboxyl group, and introduction of a functional group at the nitrogen atom of the carbazole nucleus. Through derivatization of the carprofen molecule, a series of new compounds was obtained, as presented in Table 3.

**TABLE 3:** Structural formula of new carprofen derivatives, potential COX/FAAH coinhibitors.

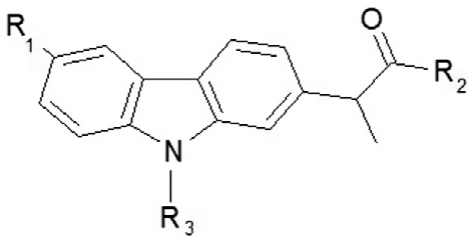			
Compd.	R_1_	R_2_	R_3_
59	‐H	‐OH	‐H
52	‐Cl	‐OCH_3_	‐H
60a	‐Cl	‐HNCH_2_CH_2_OH	‐H
60b	‐Cl	‐HNC_6_H_5_	‐H
62a	‐Cl	‐OH	‐CH_3_
62b	‐Cl	‐OH	‐CH_2_‐C_6_H_5_
62c	‐Cl	‐OH	‐CH_2_‐C_6_H_4_‐CN(4)
62d	‐Cl	‐OH	‐CH_2_‐C_6_H_4_‐Cl(4)
62e	‐Cl	‐OH	‐CH_2_‐C_6_H_4_‐OCH_3_(4)
62f	‐Cl	‐OH	‐CH_2_‐C_6_H_4_‐OCH_3_(3)
62g	‐Cl	‐OH	‐CH_2_‐C_6_H_4_‐OCH_3_(2)
64a	‐Cl	‐OH	‐SO_2_‐(CH_2_)_5_‐CH_3_
64b	‐Cl	‐OH	‐SO_2_‐C_6_H_4_‐Cl(4)
66a	‐Cl	‐OH	‐CO‐NH‐(CH_2_)_5_‐CH_3_
66b	‐Cl	‐OH	‐CO‐NH‐CH_3_
66c	‐Cl	‐OH	‐CO‐NH‐(CH_2_)_3_‐CH_3_
66d	‐Cl	‐OH	‐CO‐NH‐(CH_2_)_7_‐CH_3_
66e	‐Cl	‐OH	‐CO‐NH‐C_6_H_4_‐Cl(4)
66f	‐Cl	‐OH	‐CO‐N(CH_3_)‐(CH_2_)_5_‐CH_3_
66g	‐Cl	‐OH	‐CO‐NH‐C_6_H_11_
68	‐Cl	‐OH	‐CO‐O‐(CH_2_)_5_‐CH_3_
71a	‐Cl	‐OH	‐CO‐CH_3_
71b	‐Cl	‐OH	‐CO‐C_6_H_5_
71c	‐Cl	‐OH	‐CO‐C_6_H_4_‐Cl(4)
71d	‐Cl	‐OH	‐CO‐C_6_H_4_‐F(4)
71e	‐Cl	‐OH	‐CO‐C_6_H_4_‐OCH_3_(4)
71f	‐Cl	‐OH	‐CO‐C_6_H_4_‐Cl(3)
71g	‐Cl	‐OH	‐CO‐C_6_H_4_‐Cl(2)
71h	‐Cl	‐OH	‐CO‐C_6_H_3_‐Cl_2_(3,4)
71i	‐Cl	‐OH	oxazol‐4‐ylcarbonyl
71j	‐Cl	‐OH	1*H*‐imidazol‐4‐ylcarbonyl
71k	‐Cl	‐OH	thiazol‐4‐ylcarbonyl

**FIGURE 16:**
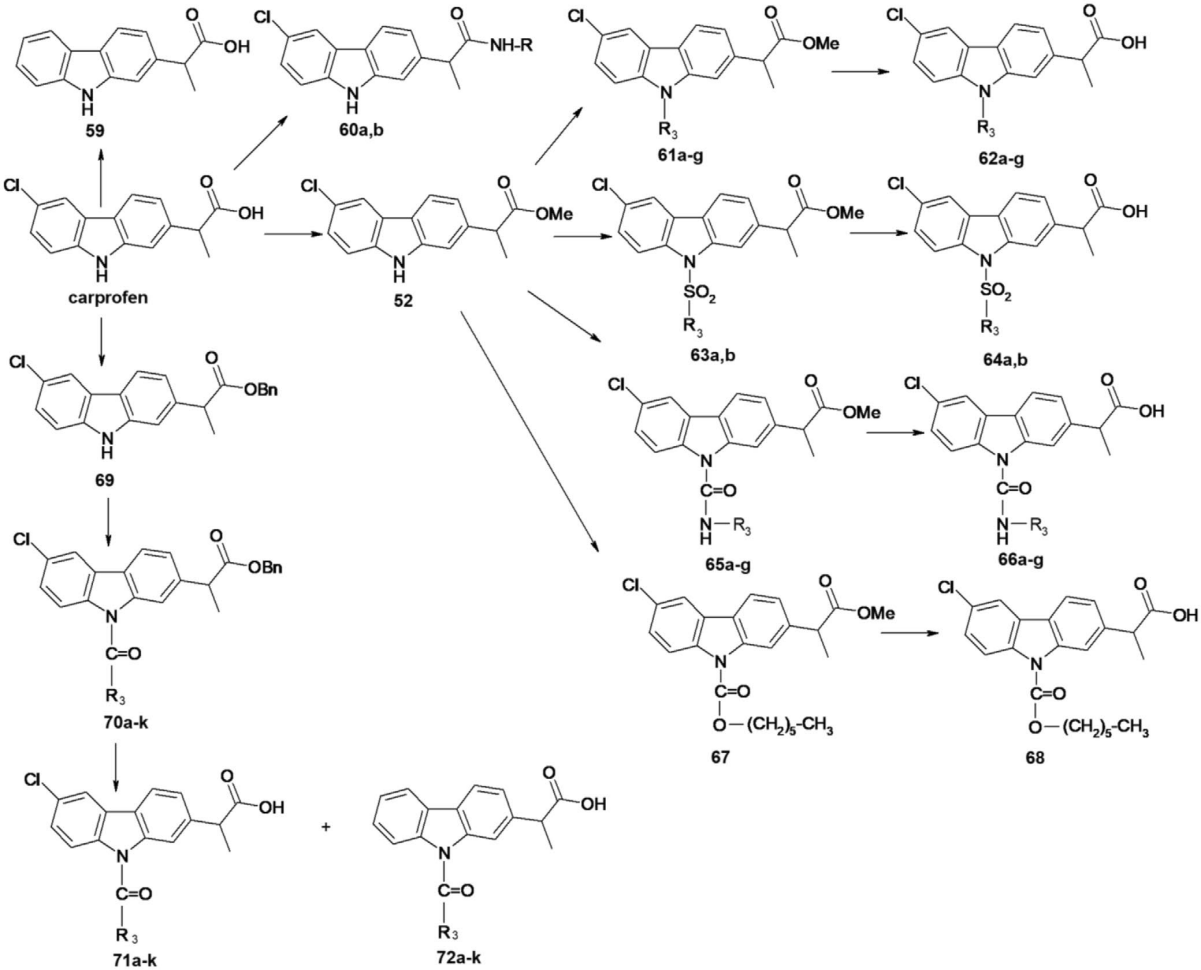
Synthesis scheme of new carprofen derivatives with COX/FAAH co‐inhibitory potential.

Figure 16 illustrates the synthesis of these compounds. Carprofen underwent hydrogenation to produce the dechlorinated derivative (59). Treating carprofen with methanol in the presence of sulfuric acid yielded methyl ester 52. Amides 60a and 60b were synthesized by reacting carprofen with ethanolamine and aniline, respectively, in pyridine with carbonyldiimidazole (CDI).

Ester 52 served as a pivotal intermediate for modifying the nitrogen atom of the carprofen molecule. N‐alkyl derivatives 61a–g were synthesized by refluxing ester 52 with various alkyl halides in CH3CN with Cs2CO3. Saponification of esters 61a–g with LiOH, followed by acid treatment, afforded the corresponding carboxylic acids 62a–g. Sulfonamide derivatives 63a,b were obtained by reacting compound 52 with sulfonyl chlorides in THF with 4‐(dimethylamino)pyridine (DMAP) and Et_3_N under thermal or microwave conditions. Saponification of the ester group yielded acids 64a,b. Urea derivatives 65a–g were obtained by microwave heating of compound 52 with various isocyanates in THF with DMAP and Et_3_N, followed by hydrolysis under basic conditions to yield acids 66a–g. Compound 66e, containing a *p*‐chlorophenyl radical, underwent hydrolysis of the methyl ester in acidic medium to avoid breaking the bond from the urea fragment, resulting in a good yield of 66e. Reacting compound 52 with hexyl chloroformate in THF with Et_3_N and DMAP yielded intermediate 67, which was hydrolyzed under acidic conditions to yield compound 68.

For acylated derivatives, the carboxyl group of carprofen was protected as *O*‐benzyl ester using benzyl bromide (BnBr), K_2_CO_3_, and DMF. Acylation reactions of benzyl ester 69 with various acyl chlorides proceeded in MeCN with DMAP and Et_3_N, yielding esters 70a–k. Debenzylation produced the corresponding acids 71a–k. H‐Cube hydrogenation equipment, under optimized conditions, was employed to reduce the formation of undesired dechlorinated derivatives 72a–j during debenzylation of compounds 71a–j. Compound 71k was obtained by using methyl ester 52 as the starting material, which was hydrolyzed in hydrochloric acid medium.

The compounds underwent testing as multitarget inhibitors aimed at concurrently blocking the activities of FAAH, COX‐1, and COX‐2 enzymes.

Carprofen displayed a fairly balanced profile in inhibiting FAAH, COX‐1, and COX‐2. Compound 59 exhibited an enhancement in FAAH potency alongside a reduction in COX‐1 and COX‐2 inhibition. It is highlighted the confirmation of the lack of COX activity in ester 52 and amides 60a and 60b, which no longer possessed a free carboxyl group with implications in COX inhibition. Conversely, compound 60b demonstrated an increase in FAAH enzyme efficiency compared to carprofen. Additionally, N‐alkylated compounds 62a–g and N‐arylsulfonyl derivatives 64a,b showed no inhibitory action on COX, yet generally displayed FAAH enzyme inhibition slightly superior to carprofen. Concerning urea derivatives 66a–g, it was noted that inhibitory activity was generally absent for both COX isoforms, while FAAH enzyme inhibitory activity relied on the type of substituent in the urea moiety, with 66a being the most potent FAAH inhibitor in this series.

Upon analysis of acylated derivatives 71a–k, it was deduced that a carbonyl function linked to the nitrogen atom of the carbazole ring might be crucial for achieving multitarget inhibition. Furthermore, the inhibitory activity on the three enzymes was markedly influenced by the nature of the heteroaromatic ring connected to the nitrogen atom of carprofen through the carbonyl group.

The most active compounds were carprofen and acyl‐derivatives 71c and 71i. Upon examining the potency of the enantiomers of these compounds, separated via chiral HPLC, it was revealed that for carprofen, the *S*‐(+) enantiomer was the sole active one against the three targets. For 71c and 71i, the *S*‐(+) enantiomer was the only one active on COX, while the *R*‐(−) enantiomer exhibited inhibitory activity on FAAH.

These FAAH/COX inhibitors hold potential as starting points for future drug discovery endeavors targeting pain, inflammation, and cancer. Additionally, they can serve as valuable tools for investigating the synergistic effects attained by simultaneously inhibiting FAAH and COX activities.

Following the same research direction, a comparative study was carried out in 2020 (Deplano et al. 2020) featuring eight compounds, amide analogues of carprofen, ketoprofen, and fenoprofen, which have been tested as FAAH/COX inhibitors.

**FIGURE 17:**
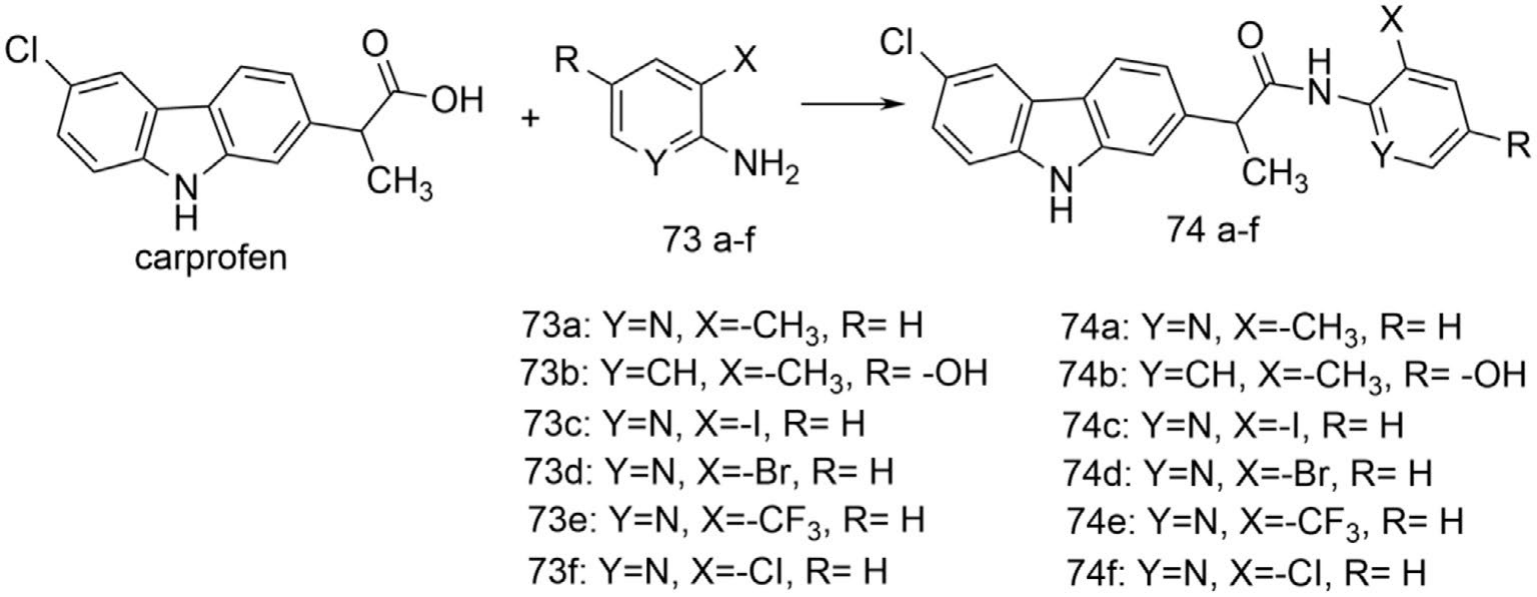
General synthetic procedure for carprofen amides 74a–f.

Coupling carprofen with substituted 2‐aminopyridines or 2‐methyl‐4‐hydroxyaniline (73a–f) resulted in amide derivatives of carprofen (74a–f). The reaction was carried out at room temperature for 36 h, using anhydrous acetonitrile as the reaction medium, in the presence of 1‐hydroxybenzotriazole hydrate and 1‐(3‐dimethylaminopropyl)‐3‐ethylcarbodiimide hydrochloride as the coupling agent (Figure 17).

2‐(6‐Chloro‐9*H*‐carbazol‐2‐yl)‐N‐(3‐methylpyridin‐2‐yl)propenamide (74a) and 2‐(6‐chloro‐9H‐carbazol‐2‐yl)‐N‐(3‐chloropyridin‐2‐yl)propenamide (74f) were found to be the most potent reversible inhibitors of 0.5 μM [^3^H]‐anandamide hydrolysis in rat brain homogenates, with IC50 values of 94 and 23 nM, respectively being 2–3 times stronger than carprofen. Both compounds inhibited the cyclooxygenation of arachidonic acid by ovine COX‐1 and were more potent inhibitors of recombinant human COX‐2 when 2‐arachidonoylglycerol was used as the substrate than when arachidonic acid was used. The results confirmed the hypothesis of the relevance of using the carprofen molecule as a template in obtaining new molecules with increased anti‐inflammatory effect and low adverse effects by simultaneously blocking COX and FAAH.

Allegretti et al. (2005) designed and synthesized CXC chemokine receptor 1 (CXCR1) ligands as novel noncompetitive inhibitors of CXCL8. These ligands have structures of 2‐arylpropionylmethanesulfonamides, (*R*)‐2‐(4‐isobutylphenyl)propionylsulfonamides, (*R*)‐2‐(4‐isobutylphenyl)propionylamides, racemic 2‐arylpropanoic acids. Chemokine CXCL8/IL‐8 has a major role in the activation of polymorphonuclear cells (PMN) at the site of inflammation.

**FIGURE 18:**
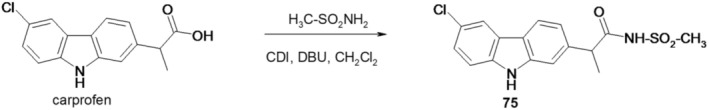
Process aimed at synthesizing derivative 75.

For the preparation of a derivative ligand of 2‐arylpropionylmethanesulfonamides, carprofen was dissolved in dichloromethane, and 1,1′‐carbonyldiimidazole (CDI) was added to this cooled solution. After the mixture was stirred for 2 h at 0°C–5°C, methanesulfonamide and diazobicyclo‐[5,4,0]undec‐7‐ene (DBU) were added and stirred for 4 h. The reaction mixture was then worked up to obtain N‐[2‐(6‐chloro‐9*H*‐carbazol‐2‐yl)propionyl]methane‐sulfonamides (75) (Figure 18).

The effect of methanesulfonamide derivative on CXCL8‐induced human PMN chemotaxis was studied, and it was found that the CXCL8 inhibitory activity was not maintained when carprofen was transformed into methanesulfonamide.

**FIGURE 19:**
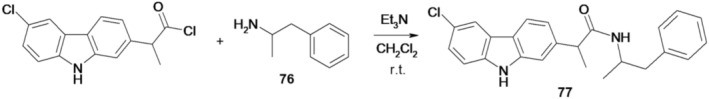
Synthesis of 2‐(6‐chloro‐9*H*‐carbazol‐2‐yl)‐*N*‐(1‐phenylpropan‐2‐yl)propanamide.

In an article published in 2023 (Manolov et al. 2023), the obtaining of new hybrid molecules between amphetamine (76) and different prophens, including carprofen (hybrids called amphens) (Figure 19), is presented, with the aim of combining the central nervous system stimulating action of amphetamine with the analgesic and anti‐inflammatory properties of prophens. The obtained amphens are characterized by their melting points, UV, NMR, and HRMS spectra. In vitro evaluation of albumin denaturation revealed significant inhibition. Thus, the IC50 value for the amphetamine‐carprofen derivative, 2‐(6‐chloro‐9*H*‐carbazol‐2‐yl)‐N‐(1‐phenylpropan‐2‐yl)propanamide (77), was 123.30 μg/mL. This indicates that the new hybrid inherits the anti‐inflammatory properties of carprofen. The toxicity of these hybrids was calculated using an in silico method.

The data are presented in LD50 values. According to calculations made using GUSAR software, the amphetamine‐carprofen hybrid demonstrates lower toxicity than standard amphetamine when administered intraperitoneally, subcutaneously, and orally. Moreover, its calculated toxicity closely resembles that of amphetamine when administered intravenously. Additionally, this study indicates that intraperitoneal administration of the hybrid offers more favorable outcomes, mitigating its toxic effects. Consequently, the hybrid exhibits promise and warrants further biological evaluations in the future.

#### 3.4.2 Carprofen Derivatives With Antimicrobial Effect

There is research that proves that some NSAIDs also have an antimicrobial effect; their mechanism of action is not yet known. An in vitro study describing the antibacterial properties on *Escherichia coli* of carprofen, bromfenac, and vedaprofen shows that these NSAIDs inhibit DNA polymerase III β subunit (Yin et al. 2014).

**FIGURE 20:**
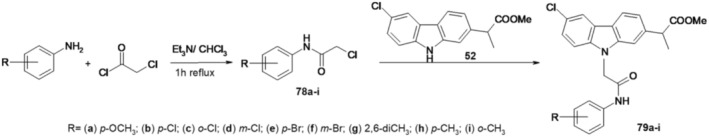
Scheme for the synthesis of compounds 79a–i.

Pattanashetty et al. (Pattanashetty et al. 2018) describe the synthesis of some *N*‐substituted carbazoles. In this sense, the 2‐chloro‐*N*‐phenylacetamide derivatives 78a–i were obtained by condensing substituted anilines with chloroacetyl chloride in the presence of triethylamine (Et_3_N), using chloroform as a solvent. These intermediates were condensed with methyl 2‐(6‐chloro‐9H‐carbazol‐2‐yl)propanoate (52), using anhydrous Cs_2_CO_3_ and acetonitrile as solvents, in order to obtain methyl 2‐(6‐chloro‐9‐(2‐oxo‐2‐(phenylamino)ethyl)‐9*H*‐carbazol‐2‐yl)propanoate derivatives 79a–i (Figure 20).

The results of the docking study showed that compounds 79g, 79i, and 79h show docking scores of 305.25, 303.43, and 281.31 kcal/mol, respectively, against the reference standard ciprofloxacin (237.66 kcal/mol), showing promising activity with low ΔG (kcal/mol) values. These compounds possess a greater inhibition capacity against the *E. coli* MurB enzyme receptor through the interaction with the active site of the receptor.

N‐substituted carbazoles showed strong antibacterial activity on strains of *Staphylococcus aureus, Bacillus subtilis*, *Escherichia coli* , and *Pseudomonas aeruginosa* , as well as anti‐inflammatory and antioxidant activity.

All tested compounds showed antibacterial activity with MICs from 0.25 to 0.8 μg/mL. The presence of the amide group and the substituent on the aromatic nucleus in the side chain of the new carbazole molecule significantly improved their antibacterial activity. In this regard, compounds 79g, 79h, and 79i, with methyl substituents on the phenyl radical, were the most active, compared to compounds containing electron‐withdrawing groups.

Compounds 79a, 79b, 79g, and 79i showed excellent anti‐inflammatory activity.

Compound 79f presented excellent antioxidant activity, and the other derivatives had moderate activity.

**FIGURE 21:**
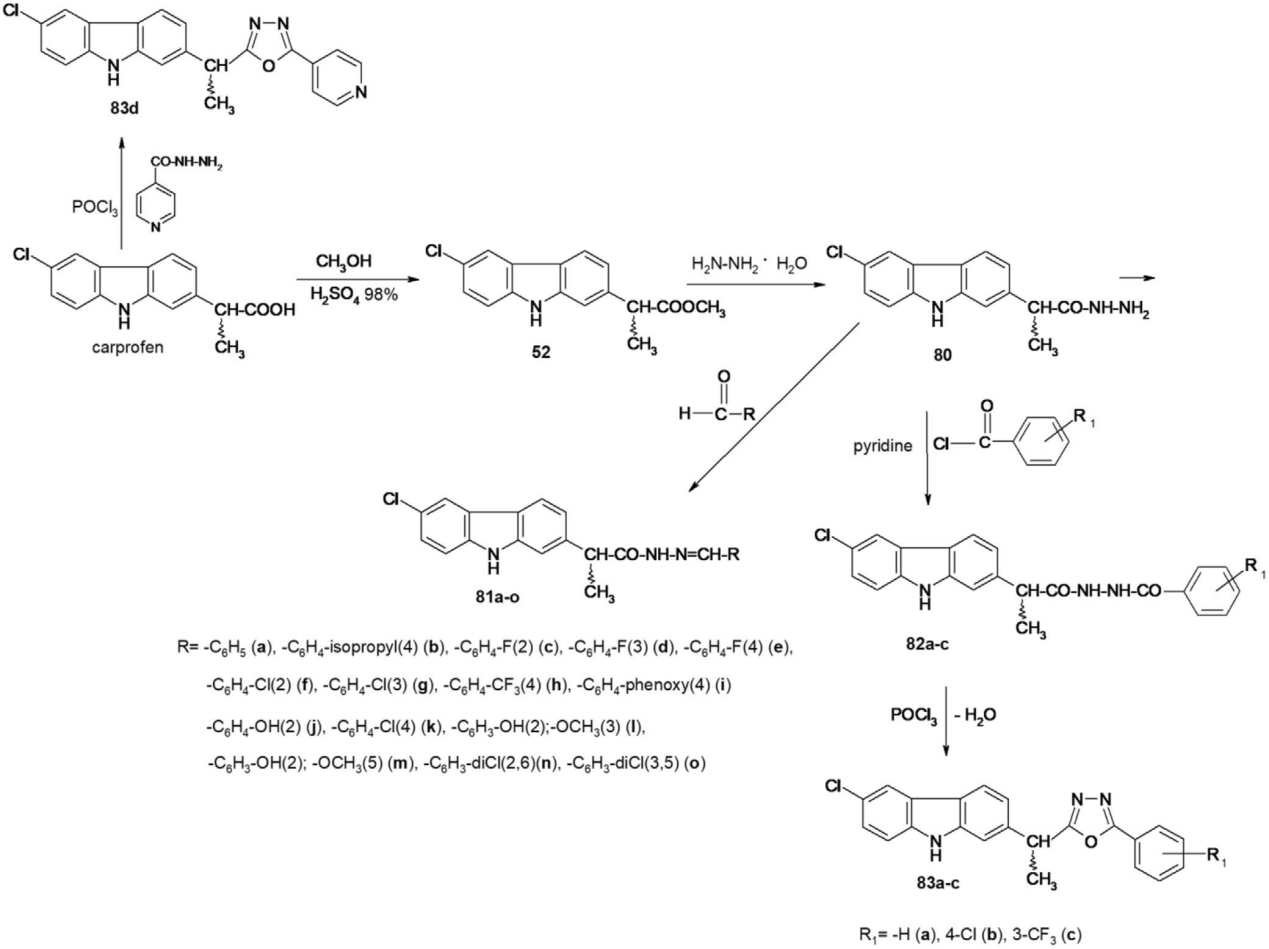
Scheme for the synthesis of new derivatives 81a–o, 82a–c, 83a–d.

New derivatives, (*EZ*)‐*N′*‐benzylidene‐(2*RS*)‐2‐(6‐chloro‐9*H*‐carbazol‐2‐yl)propanohydrazide, N‐[(2*RS*)‐2‐(6‐chloro‐9*H*‐carbazol‐2‐yl)propanoyl]‐N′‐(substituted benzoyl)hydrazine, and (*RS*)‐1‐(6‐chloro‐9*H*‐carbazol‐2‐yl)‐1‐(5‐substituted phenyl‐1,3,4‐oxadiazol‐2‐yl)ethane were synthesized according to Figure 21.

To synthesize derivatives of (*EZ*)‐N′‐benzylidene‐(2*RS*)‐2‐(6‐chloro‐9*H*‐carbazol‐2‐yl) propanohydrazide (81a–o), carprofen served as the initial material. It underwent conversion in the presence of concentrated sulfuric acid and excess absolute methanol, resulting in the corresponding methyl ester (52). Upon refluxing with 100% hydrazine hydrate in ethyl alcohol, this yielded (2*RS*)‐2‐(6‐chloro‐9*H*‐carbazol‐2‐yl)propanehydrazide, also known as carprofen hydrazide (80). Subsequently, through the reaction of compound 80 with aromatic aldehydes in absolute methanol under microwave irradiation, derivatives 81a–o were synthesized (Bordei et al. 2019; Avram et al. 2021).

By heating acylhydrazines 82a–c on a water bath with phosphorus oxychloride, 2,5‐disubstituted 1,3,4‐oxadiazoles (83a–c) were obtained.

Starting from carprofen by reaction with isoniazid in the presence of phosphorus oxychloride, (*RS*)‐1‐(6‐chloro‐9*H*‐carbazol‐2‐yl)‐1‐[5‐(4‐pyridyl)‐1,3,4‐oxadiazol‐2‐yl]ethane (83d) was also obtained.

Compounds 82a–c and 83a–c showed good antimicrobial activity, some of them being active on *E. coli* (82a—1.25 mg/mL), while others on *Candida albicans* (83c—MIC 0.625 mg/mL). The most significant result is represented by their exceptional antibiofilm activity, especially on *P. aeruginosa* biofilm (82a–c and 83a–c—MBEC 0.009 mg/mL). The cytotoxicity assay showed that at concentrations lower than 100 μg/mL, the tested compounds do not induce cytotoxicity and do not alter the cell cycle. The newly synthesized compounds exhibit drug‐like properties. ADME‐Tox profiles indicate good oral absorption and average permeability through the blood–brain barrier. However, further research is needed to reduce the predicted mutagenic potential and hepatotoxicity (Bordei et al. 2020).

The novel compounds 81j–o were evaluated against planktonic cells and biofilms of Gram‐positive *Enterococcus faecalis* ATCC 29212 and *S. aureus* ATCC 25923, Gram‐negative *P. aeruginosa* ATCC 27853 and *E. coli* ATCC 25922, and fungi *C. albicans* ATCC 10231. The compounds were more potent against microbial biofilms, exhibiting the potential to prevent the biofilm formation by *S. aureus* (81k—MBEC 0.078 mg/mL), *E. faecalis* (81k—MBEC 0.078 mg/mL) and *C. albicans* (81m—MBEC 0.009 mg/mL). The biofilms of Gram‐positive bacteria and of the fungal strain *C. albicans* were more susceptible to the compounds tested in comparison with the Gram‐negative ones. The investigated compounds could be promising in the development of novel antibiofilm agents (Bordei et al. 2022).

Compounds 82a–c and 83a–c were also investigated for their free radical scavenging properties using the free radicals 2,2′‐diphenyl‐β‐picrylhydrazyl (DPPH) and 2,2′‐azino‐bis(3‐ethylbenzothiazoline‐6‐sulphonic acid) (ABTS^•+^), and the cytotoxic capacity using a marine species, namely *Artemia franciscana*. The scavenger capacity towards DPPH free radical is lower compared to ABTS•+. Notably, compounds 82c, 82b, and 83b exhibited the highest scavenging activity. Regarding cytotoxicity, the results indicate a low level of toxicity for the tested compounds; however, further investigations are required to verify their safety profile (Bordei et al. 2021).

New derivatives of (*EZ*)‐N′‐benzylidene‐(2*RS*)‐2‐(6‐chloro‐9*H*‐carbazol‐2‐yl)propanehydrazide (81j–o) were evaluated *in silico* and for pharmacokinetic, pharmacodynamic, and pharmacogenomic properties, as well as to predict binding to therapeutic targets, by applying bioinformatics, chemical, and pharmacological computational methods, in order to identify new treatments for neurodegenerative diseases, in particular, Alzheimer's disease. Using bioinformatic tools, all new compounds, especially 81n, were observed to have drug‐like characteristics with a high potential to be used as a good candidate in the treatment of neurodegenerative disorders (Avram et al. 2021).

In recent research from 2022 (Dumitrascu et al. 2022), the synthesis of some halogenated derivatives of carprofen is presented by electrophilic substitution at the carbazole nucleus, more precisely by iodination and regioselective bromination.

**FIGURE 22:**
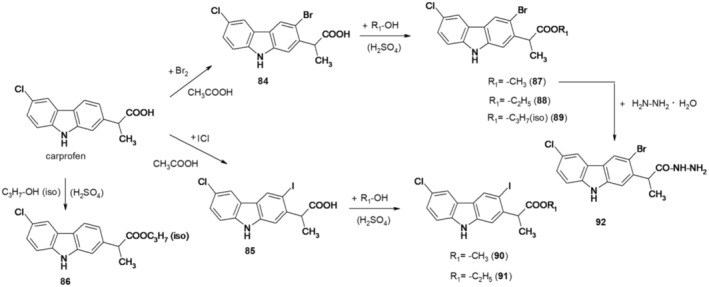
Scheme for synthesis of compounds 84–92.

Halogenation of carprofen was accomplished with bromine and iodine monochloride in glacial acetic acid. By brominating carprofen, 3‐bromocarprofen 84 was obtained, and by iodination, 3‐iodocarprofen 85. The orientation of the substitution reaction in carprofen was directed by NH and influenced by the electronic effects of the two substituents in the benzene rings. The electronic deactivating effect of the chlorine atom directed substitution in the other benzene ring at the 3‐position of carprofen. Derivatives 84 and 85 were then transformed into esters and hydrazides (Figure 22).

These compounds were characterized physicochemically and tested for their antibacterial properties on planktonic cells, but also for their ability to prevent biofilm formation. The tests were performed on Gram‐negative ( *E. coli* ATCC 25922, *P. aeruginosa* ATCC 27853) and Gram‐positive ( *S. aureus* ATCC 29213, *E. faecalis* ATCC 29212) bacteria. For three of the tested compounds, qualitative screening of antimicrobial activity revealed larger diameters of inhibition of microbial growth for Gram‐positive strains than for Gram‐negative strains. Among the compounds tested by a quantitative method, the most active were compounds 84 and 85, the most susceptible strain being *S. aureus* ATCC 25923, and the most resistant being *E. faecalis* ATCC 29212.

Compound 84, followed by compound 85, showed the most intense antibiofilm effect, especially against Gram‐positive strains, the biofilm *S. aureus* ATCC 25923 being the most susceptible to the tested compounds.

The compounds were also tested for their cytotoxic activity against HeLa cells. Some of the compounds showed a cytotoxic effect at most concentrations tested, inhibiting cell growth compared with the growth control. Cytotoxicity results also revealed a dose‐dependent cytotoxic effect against HEp‐2 cells.

With the help of in silico analysis, the pharmacokinetic and pharmacodynamic profiles of carprofen derivatives, as well as their toxicity, were determined. According to molecular docking simulations, compounds 86, 89, and 92 may be promising inhibitors of *S. aureus* .

**FIGURE 23:**
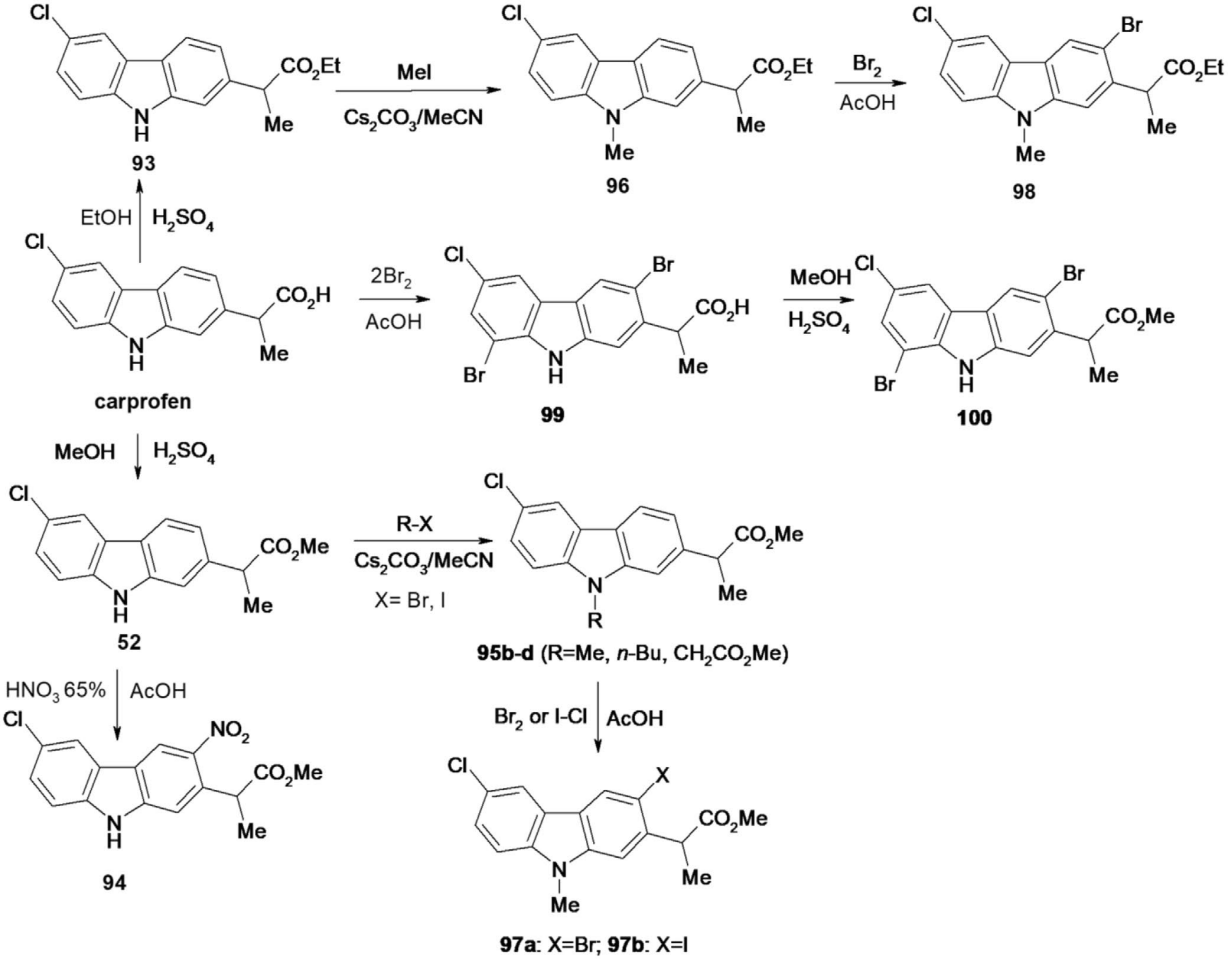
Scheme for synthesis of compounds 93–100.

In another research article (Dumitrascu et al. 2023), the synthesis of new derivatives, 94–100 (Figure 23), by nitration, halogenation, and N‐alkylation followed by halogenation of carprofen and its esters 52 and 93 is presented.

Methyl (2*RS*)‐2‐(6‐chloro‐9*H*‐carbazol‐2‐yl)propanoate (carprofen methyl ester) (52) and ethyl (2*RS*)‐2‐(6‐chloro‐9*H*‐carbazol‐2‐yl)propanoate (carprofen ethyl ester) (93) were obtained by esterification of carprofen with methanol or ethanol in the presence of H_2_SO_4_. Methyl 2‐(6‐chloro‐3‐nitro‐9H‐carbazol‐2‐yl)propanoate (94) was prepared by nitration of carprofen methyl ester dissolved in glacial acetic acid with 65% HNO_3_ at room temperature. To obtain derivatives (95b–d) and (96), esters 52 and 93 were N‐alkylated with alkyl iodides (R‐I, *R* = Me, n‐Bu) and methyl bromoacetate. By regioselective reaction of methyl 2‐(6‐chloro‐9‐methyl‐9*H*‐carbazol‐2‐yl)propanoate (95b) and ethyl 2‐(6‐chloro‐9‐methyl‐9*H*‐carbazol‐2‐yl)propanoates (96) with bromine in glacial acetic acid, the substituted 3‐bromo derivatives, methyl 2‐(3‐bromo‐6‐chloro‐9‐methyl‐9H‐carbazol‐2‐yl)propanoate (97a) and ethyl 2‐(3‐bromo‐6‐chloro‐9‐methyl‐9*H*‐carbazol‐2‐yl)propanoate (98). The substituted 3‐iodo derivative, methyl 2‐(3‐iodo‐6‐chloro‐9‐methyl‐9H‐carbazol‐2‐yl)propanoate (97b), was obtained by iodination of derivative 95b with iodine monochloride in glacial acetic acid. By regioselective electrophilic substitution, carprofen forms with two moles of bromine in glacial acetic acid, 2‐(3,8‐dibromo‐6‐chloro‐9H‐carbazol‐2‐yl)propanoic acid (99), which was easily transformed into its methyl ester 100 by esterification with methanol in the presence of H_2_SO_4_ at room temperature. The structures of these compounds were determined using NMR and IR spectroscopy. The regioselective electrophilic substitution of the carbazole ring was elucidated based on H NMR spectra. Additionally, the single crystal X‐ray structures of 99 and 100 were successfully resolved.

Evaluation of antimicrobial activity revealed that compound 99 exhibited the most promising potential, particularly against Gram‐positive strains under both planktonic and biofilm growth conditions. Compounds 94 and 100 demonstrated notable antibiofilm effects against the *P. aeruginosa* strain, along with significant antioxidant activity. Furthermore, these compounds displayed favorable oral bioavailability and showed no signs of carcinogenic or mutagenic properties. Collectively, these findings underscore the potential of novel carbazole derivatives for further exploration in the development of new antibacterial and antibiofilm agents.

In recent years, there has been a growing focus on tuberculosis, a longstanding disease that continues to claim numerous lives. Comparing with other NSAIDs (ibuprofen and the other 2‐arylpropanoic derivatives), carprofen was the most potent compound against both *M. tuberculosis* H37Rv and *M. bovis* BCG with an MIC value of 40 mg/L. The proposed mechanism of carprofen's antitubercular activity with high specificity has as target an essential protein of the *M tuberculosis* complex.

**FIGURE 24:**
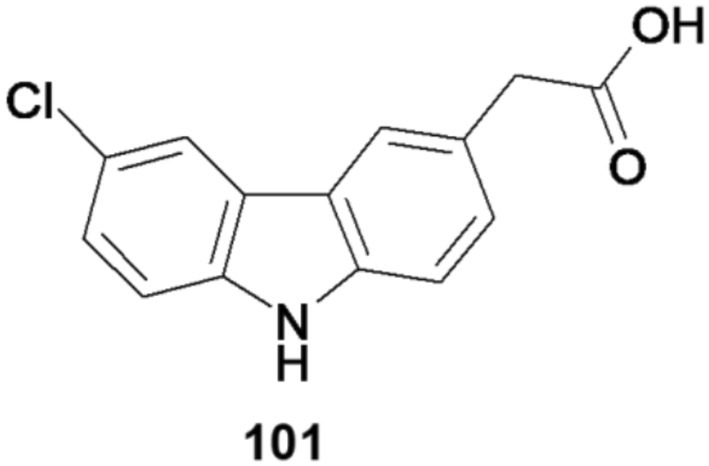
Structural formula of 2‐(6‐cloro‐9*H*‐carbazol‐3‐il)acetic acid.

Carprofen was identified to inhibit efflux pump activity in *Mycobacterium tuberculosis* (Daniel and Bhakta 2022), alter the mycobacterial biofilm phenotype, and disrupt the membrane potential of mycobacteria. Consequently, carprofen and its chemical analog, 2‐(6‐chloro‐9*H*‐carbazol‐3‐yl)acetic acid (101) (depicted in Figure 24), possess the capability to modify resistance to tuberculostatic drugs through their pleiotropic mechanisms of action. These findings suggest alternative mechanisms of action compared to current first‐line anti‐TB drugs, potentially expediting the development of new drug combinations for clinical trials (Maitra et al. 2020).

Also, the inhibition of neutrophil phagocytosis, a property that is involved in the anti‐inflammatory activity of carprofen (Tursi et al. 1982), could have major implications in the clinical treatment of tuberculosis, as the primary pathway of pathogenesis of *M. tuberculosis* requires initial phagocytic uptake of bacteria by host cells.

Carprofen and 2‐(6‐chloro‐9*H*‐carbazol‐3‐yl)acetic acid, the first of a series of new potential antimycobacterial carbazoles, offers great potential in anti‐TB therapy and therefore warrants further exploration.

In 2017 (Pattanashetty et al. 2017), a study was conducted to investigate the coumarin‐carprofen backbone. Coumarin was included in the study based on prior evidence suggesting its ability to enhance the potency of certain molecules upon integration into their structure. Additionally, being of plant origin, coumarin is readily accessible and has demonstrated a range of intriguing actions, including antimicrobial, anti‐inflammatory, antioxidant, anticoagulant, antitumor, and antiviral properties, as well as the inhibition of certain enzymes. The lactonic structure of coumarin holds significant importance due to its conjugated π‐π electron system, which facilitates bonding with potential transporters within the body. Furthermore, the molecule's geometry enables it, along with its heterocyclic derivatives, to form bonds with numerous enzymes in the body.

**FIGURE 25:**
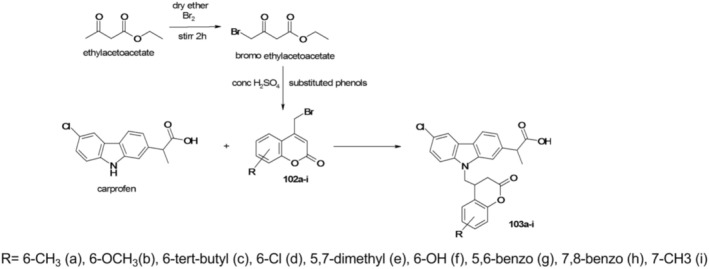
Synthesis scheme of the new coumarin‐carprofen hybrids 103a–i.

To this end, a series of coumarin‐carprofen hybrids, derivatives of the acid 2‐(6‐chloro‐9‐((2‐oxo‐2*H*‐chromen‐4‐yl)methyl)‐9*H*‐carbazol‐2‐yl)propanoic (103a–i), were synthesized through the reaction between carprofen and variously substituted 4‐bromomethyl‐2*H*‐chromen‐2‐one (102a–i). This reaction was facilitated by anhydrous potassium carbonate in acetone as the solvent. The derivatives 102a–i were in turn synthesized via Pechmann cyclization of substituted phenols with ethyl bromoacetylacetate (as depicted in Figure 25).

The new synthesized compounds were analyzed from a spectral point of view, tested to identify their action in vitro, but also through in silico docking studies.

Tests on *M. tuberculosis* strain H37Rv (ATCC strain 27,294) together with in silico molecular docking studies indicated that compounds 103a, 103f, and 103g showed promising activity. The synthesized compounds were also tested in vitro for antimicrobial and anti‐inflammatory activities. Thus, all the synthesized compounds showed excellent results with minimum inhibitory concentrations varying between 50 and 1.56 μg/mL. Compounds 103c and 103f had excellent in vitro activity on *M. tuberculosis* H37Rv, with a minimum inhibitory concentration of 1.56 μg/mL (13 times more active than pyrazinamide or ciprofloxacin, with MICs of 3.125 μg/mL mL), being promising for further investigations in an attempt to find new antituberculosis agents. Compounds 103a, 103b, 103f, and 103i, tested on *S. aureus* (MTCC 12598), *Burkholderia cepacia* (MTCC 438), *E. coli* (ATCC 25992), and *Bacillus cereus* (ATCC 11778), showed excellent antibacterial activity. Compounds 103a, 103b, 103c, and 103i had the best anti‐inflammatory activity in vitro, being evaluated by the protein denaturation method, diclofenac sodium being used as the standard drug.

In the current context related to the alarmingly increasing antimicrobial resistance and the idea of researching old molecules for new actions, carprofen has also been studied in associations. A research study from 2016 (Brochmann et al. 2016) presents the association between it and doxycycline as the most potent association in a series of 216 antimicrobial‐nonantimicrobial combinations. The association proved to be beneficial: Carprofen restored the in vitro antimicrobial susceptibility of methicillin‐resistant *Staphylococcus pseudintermedius* strains to doxycycline. Methicillin‐resistant staphylococci represent a general health problem, especially in the case of animals—infections with methicillin‐resistant *Staphylococcus pseudintermedius* (MRSP) represent a threat both to the animal itself and to the health of the population. Starting from the idea of already known combinations (e.g., amoxicillin and clavulanic acid), the need arose to look for new possible drug combinations in this sense. In conclusion, carprofen, a nonantimicrobial substance that was previously reported to have antibacterial activity in this study, in combination with doxycycline demonstrated potentiating synergism when tested on MRSP strains. Although the mechanism of action has not been fully understood, the action has been proven and may be an answer to the problem of methicillin resistance in infections with certain pathogenic germs in animals.

#### 3.4.3 Carprofen Derivatives With Antiviral Effects

The research scope of carprofen has expanded, even amid the most recent global health crisis, the COVID‐19 pandemic. The compound was singled out for investigation in a study focusing on the repositioning of existing medications for alternative purposes, notably as an inhibitor of the main protease of SARS‐CoV‐2 (M‐pro), a pivotal enzyme in virus replication. Studies have indicated a promising potential for action, which could be further enhanced through potential molecular modeling to boost its efficacy (Gimeno et al. 2020).

#### 3.4.4 Carprofen Derivatives With Anticancer Effects

In vivo, in vitro, and epidemiological studies have demonstrated that nonsteroidal anti‐inflammatory drugs (NSAIDs) appear to be effective in the chemoprevention and possibly treatment of many types of cancer (Zha et al. 2004).

Khwaja et al. tested 30 million compounds in silico and found that carprofen activates the p38 mitogen‐activated protein kinase (MAPK) leading to an increased level of the p75^NTR^ protein and the induction of apoptosis in prostate cancer cells (PC‐3 and DU‐145) and urinary bladder (T24). Further studies may lead to structurally more potent analog compounds that induce p75^NTR^‐dependent apoptosis via the p38 MAPK pathway in prostate cancer cells (Khwaja et al. 2008).

Design of prophen hybrids containing a NO donor moiety connected to an aliphatic spacer led to compounds with similar cyclooxygenase inhibition compared to their parent prophen and antiproliferative activities on PC3 cells (Bézière et al. 2008). Apart from the role played by NO and the NSAID fragment, the spacer used to connect the NO and NSAID donor group also has an active influence.

In this sense, the NO donor fragment is part of a nitrooxyalkyl group brought by ω‐bromoalkanols 104a,b; the nitric ester group was chosen for its metabolic kinetic profile. In an attempt to modify the prophene release kinetics, the aliphatic spacer was modulated.

**FIGURE 26:**
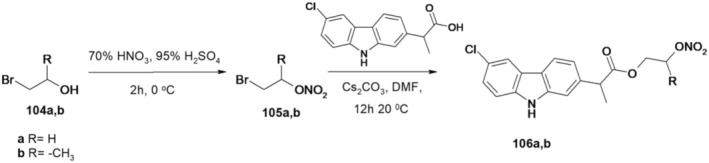
Scheme for synthesis of compounds 106a,b.

Thus, by nitration of bromoalkanols 104a,b, using a 70% HNO_3_–95% H_2_SO_4_ mixture, the corresponding nitrooxyalkyl bromides 105a,b resulted. Nitrooxyalkyl esters 106a,b were prepared by the condensation of bromides 105a,b with carprofen in the presence of Cs_2_CO_3_ (Figure 26).

The compounds were tested for their antiproliferative properties on human prostate cancer cell lines. The influence of NO release kinetics was also studied.

Using the selicistat molecule as a structural model, Mellini et al. replaced the carboxyl group of carprofen with an ester group, (substituted)amides, (substituted)hydroxamates (Mellini et al. 2012).

**FIGURE 27:**
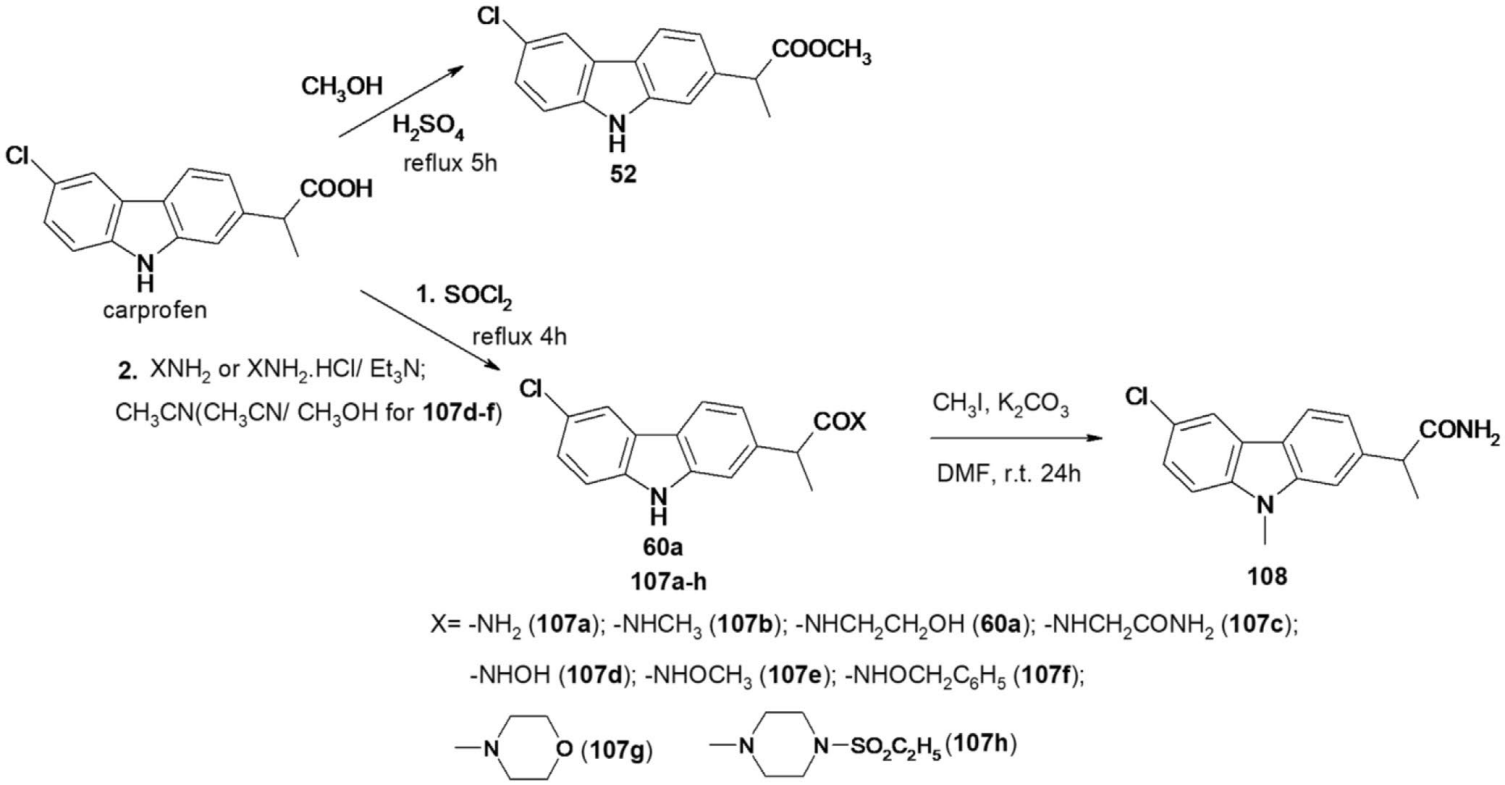
Scheme for synthesis of compounds 107a–h, 108.

Carprofen methyl ester (52) was prepared by refluxing carprofen with methanol in the presence of 96% H_2_SO_4_ as catalyst for 5 h. Reaction of carprofen with thionyl chloride afforded 2‐(6‐chloro‐9*H*‐carbazol‐2‐yl)propanoic acid chloride, which was treated with the corresponding compounds to give carboxamides 60a and 107a–h. The N‐methyl‐carbazole derivative (108) resulted upon treatment of compound 107a with iodomethane in the presence of potassium carbonate in N,N‐dimethylformamide (DMF) (Figure 27).

Compounds 52, 60a, and 107a–h were tested for SIRT1 and SIRT2 inhibitory effects.

Sirtuins (SIRT 1–7) are part of the histone deacetylase (HDAC) family and catalyze the removal of an acetyl fragment through a deacetylation mechanism dependent on NAD+. Many of the sirtuin isoforms also deacetylate nonhistone substrates such as p53 (SIRT1) and α‐tubulin (SIRT2). Sirtuins play an important role in various biological processes, including the control of gene expression, metabolism, and aging.

Compound 107a was selected to evaluate the acetylation levels of p53 and α‐tubulin in MCF‐7 breast cancer cells and human leukemia U937 cells, respectively. Carprofen and its methyl ester 52 showed weak inhibitory effects for SIRT1. Among the 6‐chloro‐9*H*‐carbazol‐2‐ylamides 107a–h and 108, the strongest SIRT1 inhibitory activity was 2‐(6‐chloro‐9*H*‐carbazol‐2‐yl)propanamide (107a) (no difference between the two enantiomers), followed by the 9‐methyl derivative 108. Compound 107a also showed the highest inhibitory activity for SIRT2 (no stereoselectivity), followed by O‐benzyl hydroxamate 107f.

Compounds 107a, 60a, 107d, 107g, and 108 were tested on the human leukemia cell line U937 to detect their effects on cell cycle progression and apoptosis induction. Carprofen and selicistat were used as reference agents, and suberoylanilide hydroxamic acid was included as a positive control for apoptosis induction. Selicistat and compound 107d arrested the cell cycle in the G1 phase, while compounds 60a, 107g, and 108 led to an increase in the number of cells in the S phase. Regarding the induction of apoptosis, unlike selicistat and carprofen, compounds 107a, 60a, and 107g induced slight apoptosis in human leukemia U937 cells, causing a small increase in DNA fragmentation.

Granulocytic differentiation was assessed by the growth of CD11c positive/propidium iodide negative cells produced by compounds 107a, 60a, 107d, 107g, and 108 in U937 cells. Histone deacetylase inhibitor entinostat (SNDX‐275 or MS‐275) was used as a positive control. No induction of granulocytic differentiation was detected under the test conditions.

These results highlight the utility of the carbazole core for the development of new SIRT1/2 inhibitors, and further studies are needed to improve the selectivity, potency, and ability to induce anticancer effects for this class of compounds.

Dong et al. presents the reductive cyanation of some halogenated derivatives using CO_2_/NH_3_ as an electrophilic CN group source (Dong et al. 2020). For the cyanation catalyzed by nickel(II) bis(acetylacetonate) [Ni(acac)_2_] the tridentate phosphine ligand, 1,1,1‐tris(diphenylphosphinomethyl)ethane (triphos), was used, resulting in the desired nitriles in good yields. Urea, a cheap and stable product, also has a promising application potential as a suitable source of CN. Mechanistic studies indicate that Ar–Ni(I) species are responsible for the C–C coupling, being involved as active silyl isocyanate intermediates. This method expands the application potential of reductive cyanation in the synthesis of nitriles, without using cyanides, which is important to safely synthesize drugs.

**FIGURE 28:**
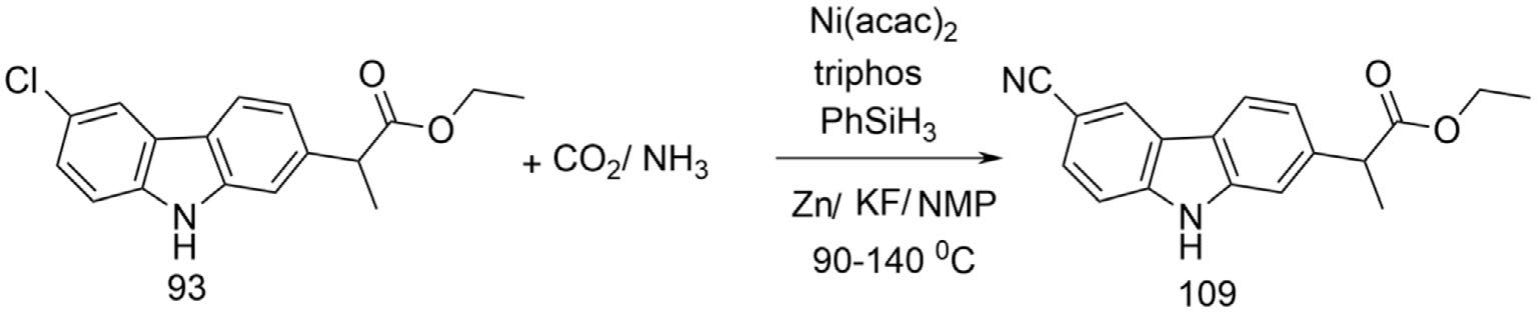
Scheme for synthesis of compound 109.

Compound 109 was obtained (Figure 28) by catalytic cyanation using CO_2_/NH_3_. Thus, under a nitrogen atmosphere, (Ni(acac)_2_), triphos, KF, Zn, and a stirring bar were introduced into a tube, which was then closed. Next, N‐methyl‐2‐pyrrolidone (NMP), carprofen ethyl ester (93) and phenylsilane (PhSiH3) were injected with a syringe. After nitrogen was removed under vacuum, CO_2_ and NH_3_ were syringed into the sealed tube. After that, the mixture was stirred for 20 h in a preheated alloyed block. After the reaction was complete, the tube was cooled to room temperature, and the pressure was carefully released.

The synthesized compound was evaluated for antiproliferative activity on MCF‐7 and Hela cells. It showed moderate antiproliferative activity on the mentioned cell lines with IC50 values of 19.35 μM (MCF‐7) and 5.91 μM (HeLa).

**FIGURE 29:**
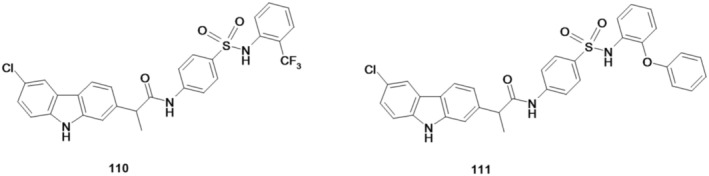
Structural formulas of compounds 110 and 111.

Huang et al. (2022) have designed, synthesized, and biologically evaluated novel arylsulfonamides with a carbazole ring, 2‐(6‐chloro‐9*H*‐carbazol‐2‐yl)‐N‐(4‐(N‐(2‐(trifluoromethyl)phenyl)sulfamoyl)phenyl)propanamide (110) and 2‐(6‐chloro‐9*H*‐carbazol‐2‐yl)‐*N*‐(4‐(*N*‐(2‐phenoxyphenyl)sulfamoyl)phenyl)propanamide (111) (Figure 29), having as a source of inspiration the natural compound sulfadixiamycin A. These derivatives were characterized by spectral analyses ^1^H NMR, ^13^C NMR, ^19^F NMR, and ESI‐MS.

The preliminary in vitro bioassay indicated that these compounds showed good cytotoxic activity on the cancer cell lines A875 (human melanoma), HepG2 (human hepatocellular liver carcinoma), and MARC145 (a subclone of African green monkey kidney cell line MA‐104) have compound 110, which has the highest inhibitory activity on all tested cell lines with IC_50_ values of 4.19 ± 0.78, 3.55 ± 0.63, and 2.95 ± 0.78 μg/mL, respectively, compared with 5‐fluorouracil.

Nevertheless, further exploration of the potential mechanisms underlying the antitumor activity of these compounds is necessary to identify potential drug candidates.

Gao et al. (2023) have designed and synthesized a series of derivatives of carbazolamides, carbazolhydrazides, and carbazolhydrazones, with the aim of improving molecular flexibility on activity.

**FIGURE 30:**
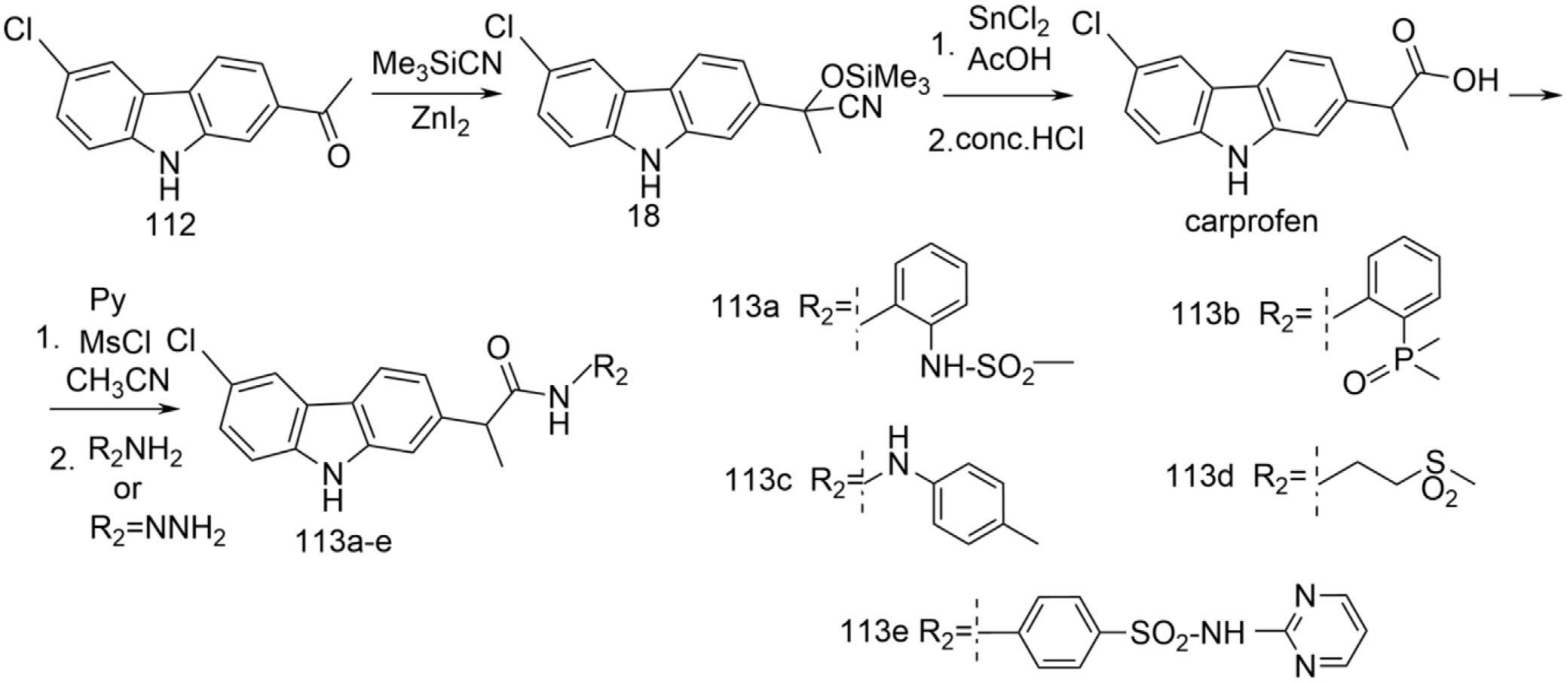
Scheme for synthesis of compounds 113a–e.

In this regard, the carbonyl group of the 2‐acetylated derivative of 6‐chloro‐carbazole (112) was treated with trimethylcyanosilane, and the resulting intermediate (18) was hydrolyzed to afford carprofen. It reacts directly with the corresponding amines and forms a series of compounds 113a–e (Figure 30).

**FIGURE 31:**
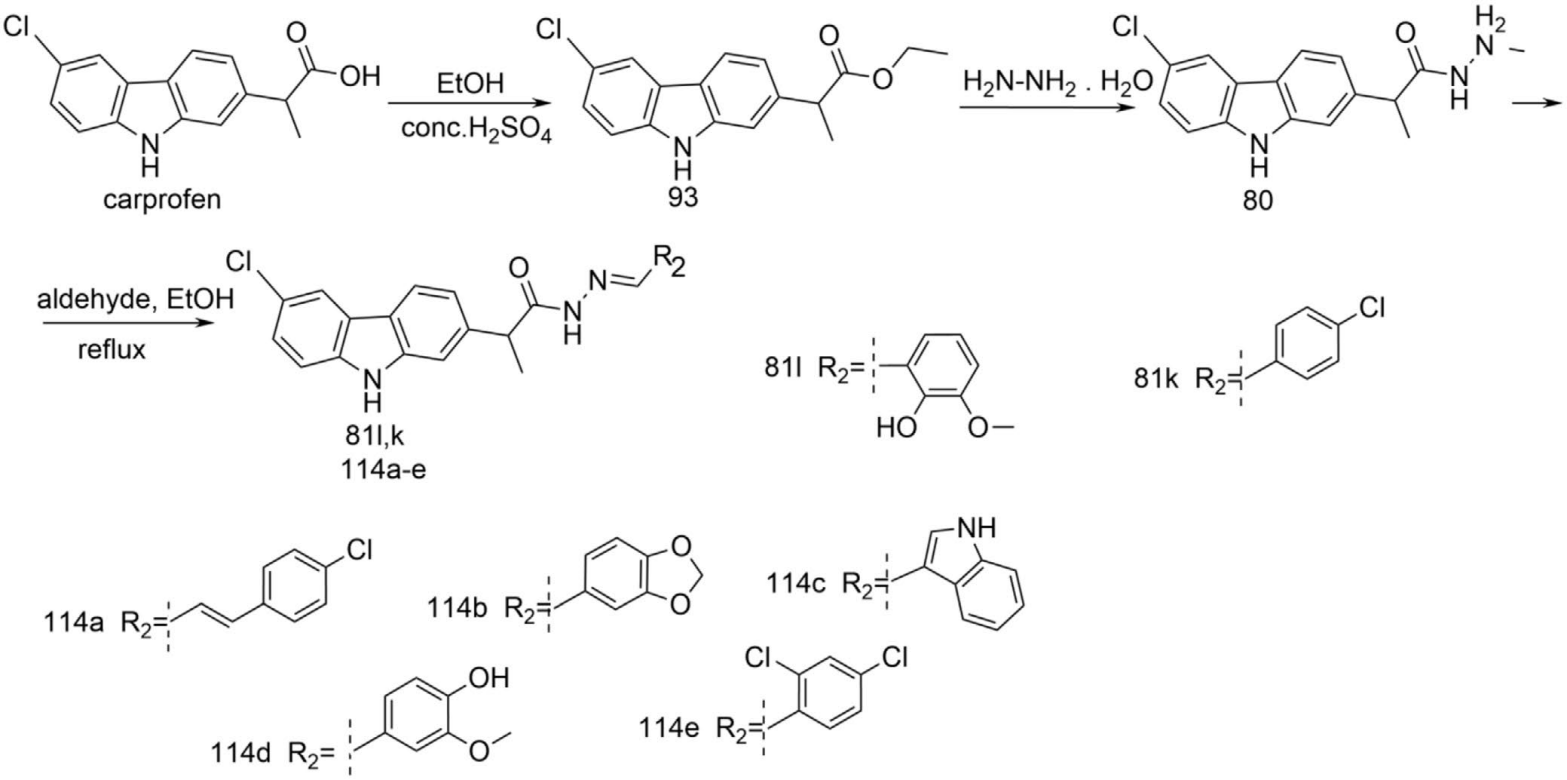
Scheme for synthesis of compounds 81l,k and 114a–e.

In the same research, carprofen was transformed into the corresponding ethyl ester (93) which, when treated with hydrazine hydrate, formed carprofen–hydrazide (80). Carprofen–hydrazide was also reacted with a carbonyl group‐bearing substrate to obtain compounds 114a–e, which link the carbazole moiety to the potentially active pharmacophore. Since the condensation method used is not stereoselective, the resulting acylhydrazone derivatives have a mixed (*E/Z*) configuration (Figure 31).

All synthesized carbazole derivatives, 113a–e, 81l,k, and 114a–e, were evaluated for their in vitro cytotoxic activity on human gastric adenocarcinoma (7901), human melanoma (A875), and African green monkey kidney (MARC145), using the classic MTT colorimetric method. The results indicated that the compound 2‐(6‐chloro‐9*H*‐carbazol‐2‐yl)‐N′‐(2‐hydroxy‐3‐methoxybenzylidene)propanehydrazide (114a) exhibited high inhibitory activities on 7901 and A875 cancer cells with lower IC_50_ values of 11.8 ± 1.26 and 9.77 ± 8.32 μM, respectively, which could be a starting point for the discovery of new anticancer agents with a carbazole core.

In an attempt to treat aggressive tumors such as triple‐negative breast tumors (TNBC), novel bifunctional molecules with selective antiproliferative activity and efficient small interfering RNA (siRNA) transfection capabilities are being designed and developed by targeting a certain gene. Cationic lipids are an option for siRNA delivery due to their ability to attach to negatively charged membranes and trigger siRNA uptake.

Due to their ability to deliver siRNA and their intrinsic anticancer properties, cationic lipid therapies have become valuable in the treatment of malignant cancers.

Thus, Vaidya et al. (2023) have synthesized a series of acid‐containing cationic lipids and evaluated bifunctional activity for their anticancer activity and survivin and RNA‐mediated anticancer activity.

**FIGURE 32:**
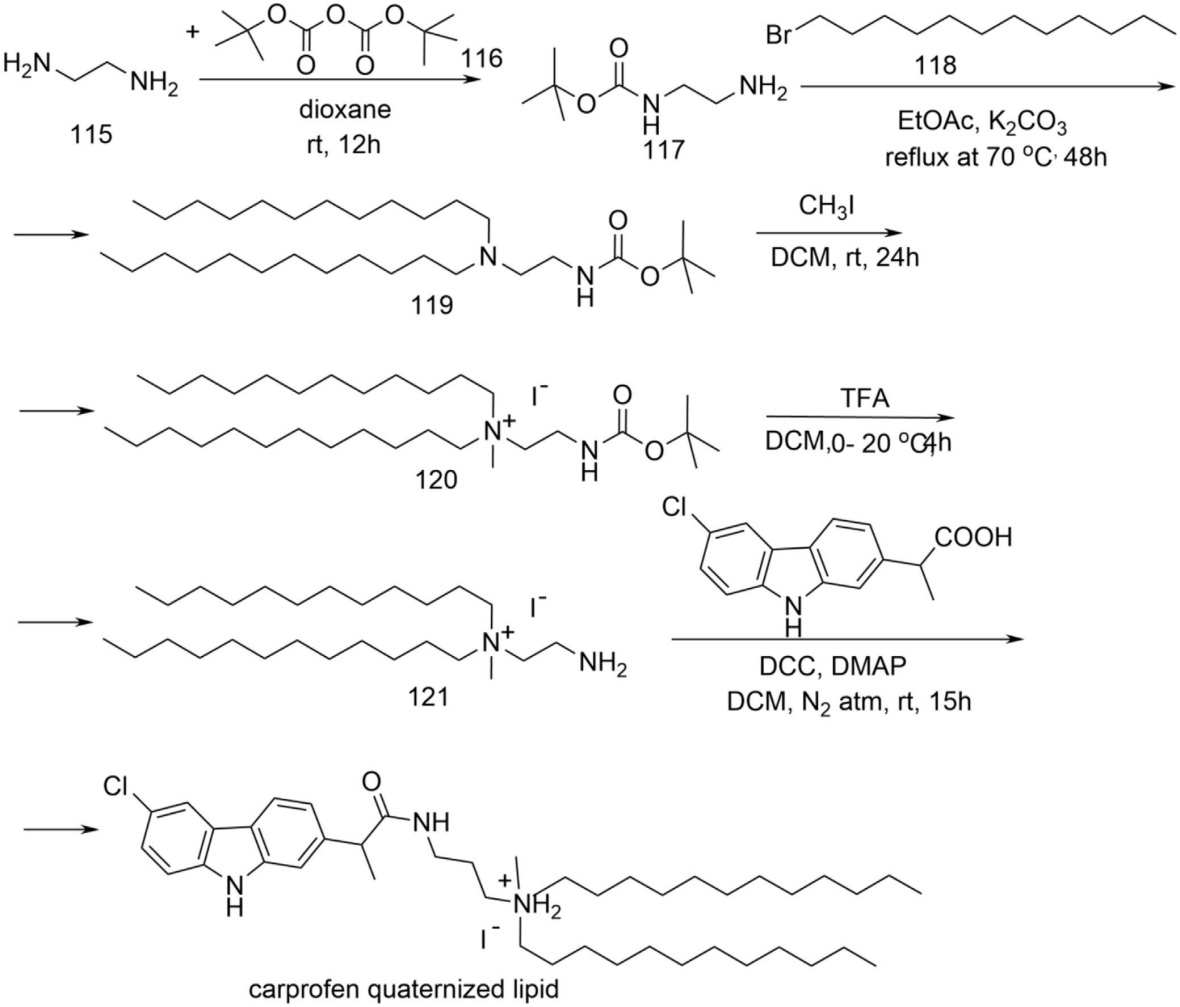
Scheme for synthesis of carprofen quaternized lipid.

Ethylenediamine (115) and di‐tert‐butyl dicarbonate (116), in dioxane medium, were stirred for 12 h at room temperature, resulting in tert‐butyl‐(2‐aminoethyl)carbamate (117). The mixture of compound 117 in anhydrous ethyl acetate, K_2_CO_3_, and 1‐bromododecane (118) was refluxed at 70°C for 48 h, yielding the intermediate tert‐butyl (2‐(dodecylamino)ethyl)carbamate (119). Compound 119, dissolved in dichloromethane (DCM), was stirred at room temperature for 24 h with methyl iodide, forming N‐(2‐((tert‐butoxycarbonyl)amino)ethyl)‐N‐dodecyl‐N‐methyldodecane‐1‐aminium (120). Over intermediate 120 dissolved in DCM, trifluoroacetic acid (TFA) was added dropwise under nitrogen atmosphere at 0°C, and then the reaction mixture was stirred at room temperature to completely deprotect and remove the tertiary group butyloxycarbonyl (BOC), resulting in a quaternary amine intermediate (121). Compound 121 and 4‐dimethylaminopyridine (DMAP) were added over a solution of carprofen dissolved in DCM. N,N′‐dicyclohexylcarbodiimide (DCC) was added to this mixture under a nitrogen atmosphere. Then, the reaction mixture was heated to room temperature and stirred for 15 h, and after the completion of the reaction, carprofen quaternized lipid resulted (Figure 32).

The procedures are identical for obtaining the other quaternized lipids, ketoprofen lipid, ibuprofen lipid, etodolac lipid, naproxen lipid, indomethacin lipid, and mefenamic acid lipid. The seven cationic lipids were characterized using IR, NMR, and HRMS.

In the MTT cell viability assay on 4T1 cell lines, etodolac lipid (Etodo) and mefenamic acid lipid (Mef) showed increased cytotoxicity at a lower concentration levels, and therefore these lipids were used for further generation of liposomes and lipoplexes.

Cationic liposomes were obtained by generating a thin film using the synthesized cationic lipids and dioleoyl‐3‐trimethylammonium propane (Dotap) and cholesterol (Chol) (as colipids) to create the nonviral vector for siRNA delivery. To further enhance the efficacy of these liposomes, they were used to create lipoplexes by incubating the siRNA and liposomes for 1 h at 37°C.

New cationic liposomes Etodo: Dotap, Etodo: Chol, Mef: Dotap, Mef: Chol and cationic lipoplexes Etodo: Dotap &RNA, Etodo: Chol & siRNA, Mef: Dotap & siRNA, Mef: Chol & siRNA are characterized.

The test results showed that lipoplexes with Etodo:Dotap‐siRNA and Mef:Dotap‐siRNA showed a homogeneous particle size and a positive zeta potential. In addition, biological investigations highlighted improved siRNA delivery, improved transfection efficiency, and anticancer activity. Etodo: Dotap & siRNA and Mef: Dotap & siRNA lipoplexes destroy the survival gene in the human lung cancer cell line (A549) and mouse triple‐negative breast cancer cell line 4T1, improve apoptosis, and produce the arrest of the G1 or G2/M cell cycle phase in both cell types. In vivo results showed that treatment with lipoplexes significantly reduced tumor growth and tumor weight compared to the control. Thus, it is expected that the new liposome formulations based on quaternary amines will open new perspectives in the development of siRNA‐based technology and anticancer therapy.

The carprofen–Pt(IV) and (carprofen)_2_–Pt(IV) complexes were prepared and tested through primary in vitro antitumor screening, observing that the second complex exhibits superior properties and was selected to obtain nanoparticles. The nanoparticles NPs@(carprofen)_2_–Pt(IV) were prepared by encapsulating (carprofen)_2_–Pt(IV) in DSPE‐PEG2000. Transferrin‐modified carprofen platinum(IV) nanoparticles, Tf‐NPs@(carprofen)_2_–Pt(IV), were obtained by modifying the surface of NPs@(carprofen)_2_–Pt(IV) nanoparticles with transferrin protein.

**FIGURE 33:**
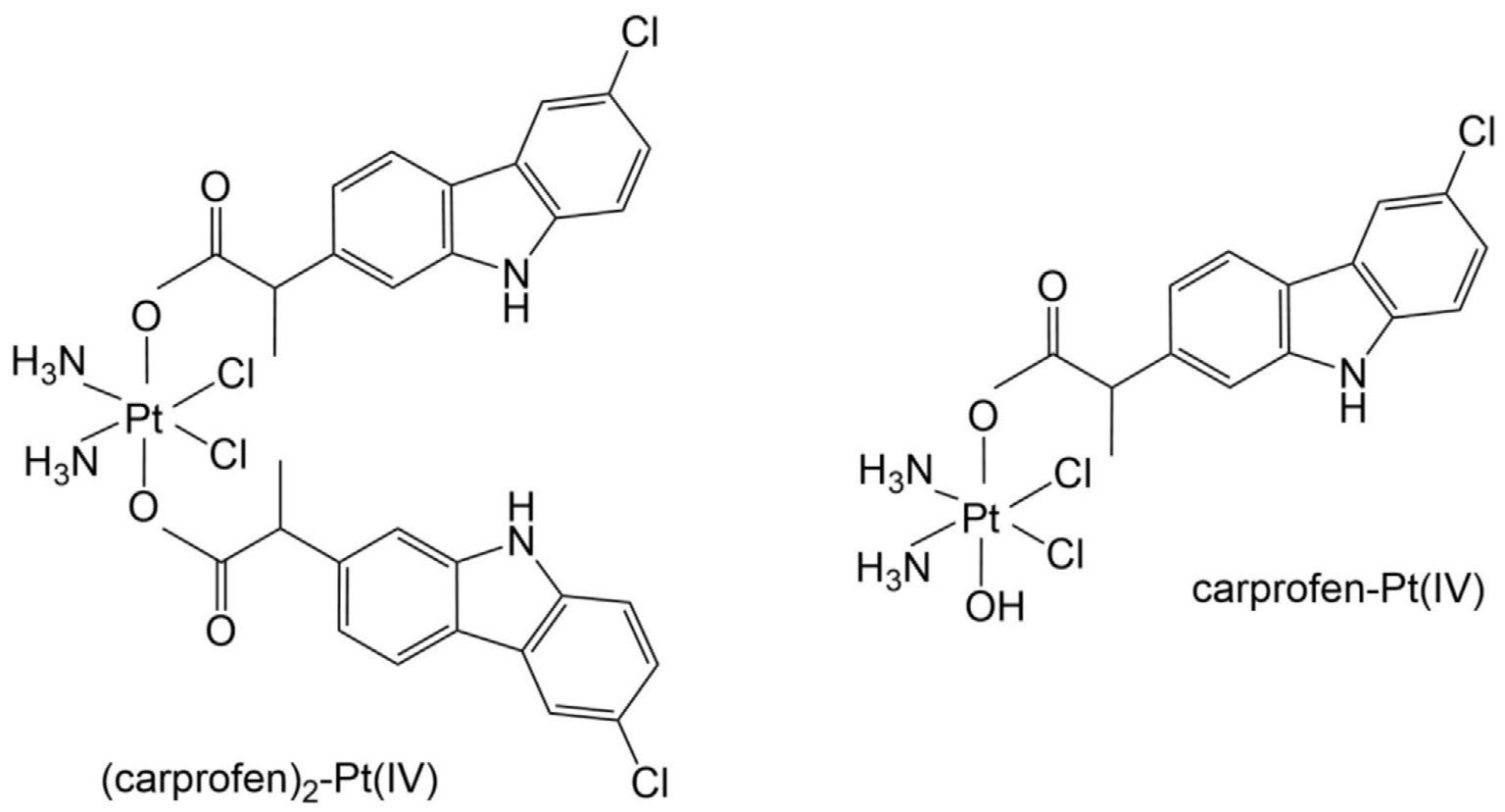
Carprofen–Pt(IV) and (carprofen)_2_–Pt(IV) complexes.

Subsequently, the antitumor activity and antimetastatic properties of NPs@(carprofen)_2_–Pt(IV) and Tf‐NPs@(carprofen)_2_–Pt(IV) were evaluated both in vitro and in vivo. The likely mechanisms of action of these nanoparticles were also studied. Tf‐NPs@(carprofen)_2_–Pt(IV) exhibited superior pharmacokinetic profiles and reduced side effects compared to the carprofen−Pt(IV) complexes. The transferrin protein enhanced the tumor targeting properties and, consequently, the antitumor potency. Tf‐NPs@(carprofen)_2_–Pt(IV) demonstrated effective antiproliferative and antimetastatic activities with reduced toxicity. The results obtained are significant for future efforts aimed at developing new nonsteroidal anti‐inflammatory drugs–platinum(IV) for the treatment of metastases (Zhang et al. 2024) (Figure 33).

#### 3.4.5 Carprofen Derivatives With Anti‐Alzheimer's Effect

Narlawar et al. (2006) synthesized *N*‐sulfonylated and *N*‐alkylated derivatives of carprofen, which were then investigated for inhibition and modulation of gamma‐secretase, a proteolytic complex involved in Alzheimer's disease that cleaves the transmembrane domain of β‐amyloid precursor protein until it is sufficiently reduced to allow the release of β‐amyloid from the membrane. The introduction of a lipophilic, arylsulfone, or alkyl substituent transformed the COX‐2 inhibitor carprofen into a potent gamma‐secretase modulator. Lipophilic substituents determine amphiphilic properties for carboxylic acids, which can interact with membranes.

**FIGURE 34:**
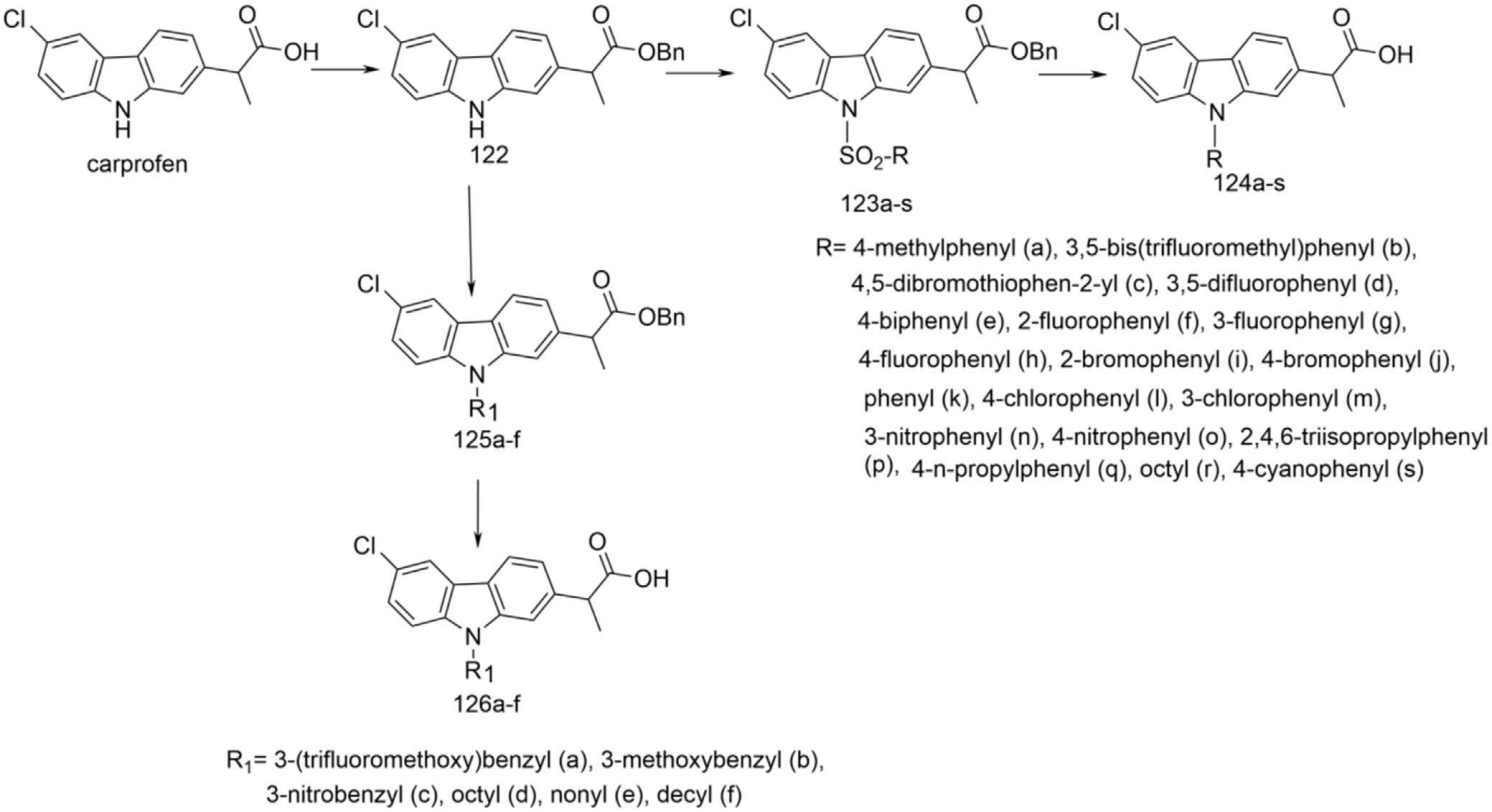
Scheme for synthesis of compounds 124a–s and 126a–f.

These derivatives were obtained according to Figure 34.

Thus, the carboxyl group of carprofen was protected by esterification with benzyl bromide in DMF to give the benzyl ester (122), which was N‐sulfonylated using NaH and a corresponding sulfochloride (R‐SO_2_‐Cl) in THF. The benzyl group of compound 123 was removed by hydrogenation (for compounds 123a–m, p–r) or basic hydrolysis (123n–o, s), yielding 124a–s.

Esters 125a–f were obtained from benzyl ester 122, alkyl halides, and NaH in THF. By hydrogenation of the corresponding ester (125a,b,d–f), or hydrolysis in basic medium for 125c, the N‐alkylated derivatives of carprofen 126a–f were prepared.

The most potent compounds, namely 2‐(6‐chloro‐9‐(2,4,6‐triisopropylphenylsulfonyl)‐9*H*‐carbazol‐2‐yl)propanoic acid (124p) and 2‐(6‐chloro‐9‐decyl‐9*H*‐carbazol‐3‐yl)propanoic acid (125f), induced the selective reduction of Aβ42 levels while promoting an increase in the less aggregation‐prone Aβ38.

Although some of the *N*‐sulfonylated derivatives of carprofen exhibit potency comparable to the best *N*‐alkylated analogs, their 50% increase in topological polar surface area suggests a lower likelihood of penetrating the blood–brain barrier. Consequently, *N*‐alkylated derivatives are preferred over *N*‐sulfonylated derivatives for further investigation.

#### 3.4.6 Other Research Directions Regarding Carprofen

The presence of the carboxyl group in the structure of carprofen allows the formation of salts with organic bases with nitric oxide‐enhancing properties, which is an interesting research direction pursued by Garvey D et al. The organic nitric oxide‐enhancing compounds include organic nitrates, nitrites, nitrosothiols, thionitrates, NONOates, heterocyclic nitric oxide donors, and/or nitroxides. Thus, carprofen's anti‐inflammatory effects are enhanced, with NO acting as an additional mediator of inflammation and pain relief. Additionally, the gastrointestinal side effects of carprofen can be reduced by NO, as a vasodilator, by promoting blood flow to the gastric mucosa and enhancing the production of protective mucus. NO‐releasing compounds may also influence the rate of drug absorption and the controlled release of the active ingredient. This could lead to more efficient delivery of carprofen to target tissues, reducing fluctuations in drug concentrations and providing more stable pain relief (Garvey and Lew 2006).

To improve the pharmacokinetic profile and bioavailability of carprofen, the preparation of cocrystallized compounds can be considered. In this regard, Bruni et al. obtained cocrystals of carprofen with 4,4′‐dipyridyl using the solvent evaporation and wet/dry grinding methods. Two different crystal structures (triclinic or monoclinic cells) were obtained depending on the molar ratio (Bruni et al. 2012).

Another approach is that of nano‐hybrid compounds that combine carprofen with inorganic hosts such as layered double hydroxide (LDH). Capsoni et al. synthesized the Carprofen‐Zn2Al‐LDH hybrid compound by the coprecipitation method, using carprofen, zinc nitrate, and aluminum nitrate in a molar ratio of carprofen:Zn:Al 2:2:1. In this way, the release rate of carprofen was improved, and the reduction in gastric irritation was also anticipated due to the antiacid effect of LDH (Capsoni et al. 2018).

Recent attention has been focused on the development of poly(*D,L*‐lactide‐co‐glycolide) acid nanoparticles for the controlled delivery of carprofen at the dermal, intra‐articular levels in rabbits, as well as for their application on porcine mucous membranes and ophthalmic tissues, as well as human, porcine, and bovine skin, for use as an anti‐inflammatory agent (Parra‐Coca et al. 2021; Gómez‐Segura et al. 2020; Parra et al. 2015, 2016).

The results indicate that carprofen nanoparticles offer significant advantages in most tissues. They are more effective and safer than carprofen solution and do not alter tissue structure. This suggests great potential for local treatment of various inflammatory diseases in pigs or humans. This approach also minimizes the side effects typically associated with NSAIDs (Gómez‐Segura et al. 2020).

Permeation studies showed similar diffusion values between human and porcine skins, with higher values observed for bovine skin and no irritation on rabbit skin. The carprofen‐containing nanoparticles are of interest in the dermal treatment of local inflammation (Parra et al. 2016).

They also have good solubility, making them suitable for intraocular administration, and testing on isolated rabbit cornea demonstrated that an adequate amount of carprofen was retained in the tissue, avoiding excessive permeation and potential systemic levels. Furthermore, no ocular irritation was observed. The in vivo ocular anti‐inflammatory efficacy test confirmed the optimal effectiveness of the carprofen‐containing nanoparticles and their potential application in eye surgery (Parra et al. 2015).

## 4 Concluding Remarks and Future Perspectives

Through a thorough review of the specialized literature, we have highlighted that the carprofen molecule continues to captivate the interest of researchers. It remains a valuable starting point for drug design and repurposing studies. The development of novel carprofen derivatives with equal or superior activity presents an exciting avenue for further exploration. Such derivatives could potentially offer enhanced anti‐inflammatory, antimicrobial, antimycobacterial, anticancer, and anti‐Alzheimer's properties. Additionally, emerging molecular development strategies, particularly those involving artificial intelligence‐assisted techniques, provide new opportunities to expand the therapeutic potential of carprofen in order to develop new compounds with more targeted therapeutic profiles.

## Conflicts of Interest

The authors declare no conflicts of interest.

## Data Availability

The data that support the findings of this study are openly available in [repository name] at [DOI], reference number [reference number].
